# Proteochemometric Modeling of the Bioactivity Spectra of HIV-1 Protease Inhibitors by Introducing Protein-Ligand Interaction Fingerprint

**DOI:** 10.1371/journal.pone.0041698

**Published:** 2012-07-27

**Authors:** Qi Huang, Haixiao Jin, Qi Liu, Qiong Wu, Hong Kang, Zhiwei Cao, Ruixin Zhu

**Affiliations:** 1 School of Life Sciences and Technology, Tongji University, Shanghai, People's Republic of China; 2 Key Laboratory of Applied Marine Biotechnology Ministry of Education, Ningbo University, Ningbo, People's Republic of China; 3 School of Pharmacy, Liaoning University of Traditional Chinese Medicine, Dalian, Liaoning, People's Republic of China; 4 Institute for Advanced Study of Translational Medicine, Tongji University, Shanghai, People's Republic of China; Centro de Biología Molecular Severo Ochoa (CSIC-UAM), Spain

## Abstract

HIV-1 protease is one of the main therapeutic targets in HIV. However, a major problem in treatment of HIV is the rapid emergence of drug-resistant strains. It should be particularly helpful to clinical therapy of AIDS if one method can be used to predict antivirus capability of compounds for different variants. In our study, proteochemometric (PCM) models were created to study the bioactivity spectra of 92 chemical compounds with 47 unique HIV-1 protease variants. In contrast to other PCM models, which used Multiplication of Ligands and Proteins Descriptors (MLPD) as cross-term, one new cross-term, *i.e.* Protein-Ligand Interaction Fingerprint (PLIF) was introduced in our modeling. With different combinations of ligand descriptors, protein descriptors and cross-terms, nine PCM models were obtained, and six of them achieved good predictive abilities (Q^2^_test_>0.7). These results showed that the performance of PCM models could be improved when ligand and protein descriptors were complemented by the newly introduced cross-term PLIF. Compared with the conventional cross-term MLPD, the newly introduced PLIF had a better predictive ability. Furthermore, our best model (*GD & P & PLIF: Q^2^_test_ = 0.8271*) could select out those inhibitors which have a broad antiviral activity. As a conclusion, our study indicates that proteochemometric modeling with PLIF as cross-term is a potential useful way to solve the HIV-1 drug-resistant problem.

## Introduction

Acquired immunodeficiency syndrome (AIDS), caused by human immunodeficiency virus (HIV), is one of the most fatal diseases to threat human life for its infectivity and high mortality. Since its recognition in 1981, more than 60 million people have been infected with HIV around the world, and approximately 25 million people have died of AIDS. Nowadays, more than 34 million are living with HIV infection [1], [2]. Currently, the main strategies for treating AIDS are through disrupting one or several key steps of HIV life cycle to control the replication rate of HIV virus.

HIV-1 protease is one of the main therapeutic targets in HIV and it is a dimeric protein composed of two identical 99-residue chains. The protease cleaves the Gag-Pol polyprotein into structure proteins and enzymes, which is a necessary step for the generation of new infectious virus particles, and nine of the twenty-eight FDA-approved anti-HIV drugs in current use target the HIV-1 protease. However, mutations were found in the protease soon after the HIV protease inhibitors were introduced, and the high mutation rate of HIV-1 protease allows the virus to escape from the antiviral therapy [3]. So it is necessary to acquire a reasonable method to predict antivirus capability of compounds for a wide spectrum of HIV.

To date, for experimental methods, high-throughput screen is mostly used to filter novel compounds against all kinds of targets as well as HIV mutated variants; for *in silico* methods, molecular docking [4], [5], [6], pharmacophore models [7], [8], quantitative structure-activity relationship (QSAR) [6], [9], [10], [11] *etc* are widely used to virtually screen antiviral compounds against HIV mutated variants. However, these methods are limited to the study of the molecular recognition of one series of ligands interacting with single target. In addition, the experimental assays are not only cost-consuming but also limited by the repertoire of compounds [12]. What the previous methods obtained are only suitable for single variant rather than an overall bioactivity profile of compounds' activity against series of variants. Although several methods have been proposed on multi-target, like Liu et.al [13], [14]_ENREF_13_ENREF_13 applied multi-task learning in QSAR to analyze and design the novel multi-target HIV-1 inhibitors as well as HIV-HCV co-inhibitors; Ragno et.al [5], De Martino et.al [15] and Sotriffer et.al [16] used cross-docking to gain insight on the mode of action of new anti-HIV agents against both wild-type and resistant strains, in such multi-target QSAR models, there are no explicit descriptions for targets, especially for the interaction information of target-ligand pairs [13], [14]. On the other hand, it is well known that docking is time-consuming, and the accuracy and versatility of the scoring functions are the main issues for the current docking algorithms [17], [18], [19], [20], [21].

More recently, proteochemometric modeling has been widely used to study the mechanisms for molecular recognition of series of proteins, and widely applied in multiple variants- [22], [23], [24], superfamily- [25], [26], kinome- [27], as well as proteome-wide interaction [28], [29], [30]. This method combines both the ligand and target descriptors, and then correlates them to the activity data. Therefore, PCM models can be considered as an extension of the QSAR models, which are only based on the ligand information. So far proteochemometrics have been successfully applied to HIV-1 protease [23], [24] and reverse transcriptase [22] to analyze drug resistance over the mutational space for multiple variants and multiple inhibitors.

However, in most of previous proteochemometric modeling, cross-terms were derived from Multiplication of Ligand and Protein Descriptors (MLPD) [23], [24], [25], [26], [31]. Cross-term is an additional introduced term. Although it was introduced to account for the complementarity of the properties of the interacting entities and it can describe the two entities simultaneously, the significance is not easy to understand. In addition, a lot of descriptors will be generated by MLPD so that it is computationally time-costive and with much redundancy. To address this issue, here we presented a new cross-term protein-ligand interaction fingerprint (PLIF) [32], [33], [34], [35], which describes the interaction of a protein's residues with its ligand. In our study, we used PLIF to construct PCM models to analyze bioactivity profiles of series of inhibitors against series of HIV-1 protease variants comprehensively.

## Results and Discussion

### Kernel Selection

Our PCM modeling was performed based on support vector regression (SVR). To select an effective kernel function for SVR, 10-fold cross-validation was first performed based on all the data set with all the four kernel functions in choices. The results of Q^2^_CV_ of each model with different combinations of descriptor blocks were listed in **Table 1**. From the table, the results show that most of the models run with Normalized Poly Kernel function obtained better predictive ability than those with the other three kernel functions. The paired *t*-test also showed that Normalized Poly Kernel function was more suitable for this dataset in PCM modeling (*p*-values are 0.0006827, 8.652e-06, 0.0301, compared with Poly Kernel function, Puk function and RBF Kernel function respectively). Therefore, **Normalized Poly Kernel** function was selected here.

**Table 1:** Q^2^_CV_ of each model with different combinations of descriptor blocks.

Models with different descriptor combinations	Normalized Poly Kernel	Poly Kernel	Puk	RBF Kernel
GD×P	***0.6429***	0.3643	0.1586	0.2988
DLI×P	***0.6327***	0.2054	0.2511	0.4221
PLIF	***0.5572***	0.5727	0.1627	0.5475
GD & P & GD×P	***0.6476***	0.3615	0.1581	0.2916
GD & P & PLIF	***0.7022***	0.3572	−0.0214	0.6988
GD & P	***0.6623***	0.5702	0.2759	0.6731
DLI & P & DLI×P	***0.6273***	0.2243	0.2509	0.4155
DLI & P & PLIF	***0.6731***	0.3475	0.0306	0.6880
DLI & P	***0.6095***	0.5195	0.3831	0.6544

Support vector regression has a number of advantages over the conventional linear regressions, especially for its robustness to avoid overfitting [28], [36], [37]. By the use of the non-linear kernel, SVM projects the data into a high-dimensional feature space and correlation is then performed in this hyperspace. The selection of the kernel function for SVR is very important because we may construct learning machines based on how this inner-product kernel is generated. The four kernels (summarized in **Table 2**) are implemented in SMOreg of Weka and commonly used in support vector machine. In previous SVM classification studies, experiments were carried out using two to four of these kernels for comparison. In different studies, different kernel was adapted [38], [39], [40]. Therefore, a kernel that performs well on one dataset does not necessarily perform well on another one. In our regression analysis, Normalized Poly Kernel indicated the best predictive ability among others.

**Table 2:** Summary of Kernels.

Type of Kernels	Functions
Normalized Poly Kernel	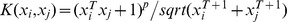
Poly Kernel	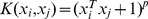
Puk	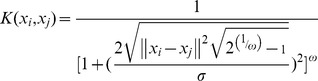
RBF Kernel	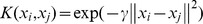

### Development and evaluation of the PCM models

With the selected Normalized Poly Kernel function, nine PCM models with different descriptor combinations were created from all the datasets. 20 ligand-protein pairs behaved as outliers (Z-score> = 2.0 in no less than five of these nine models) , thus they were removed (see **Table S3**).

**Figure 1:**
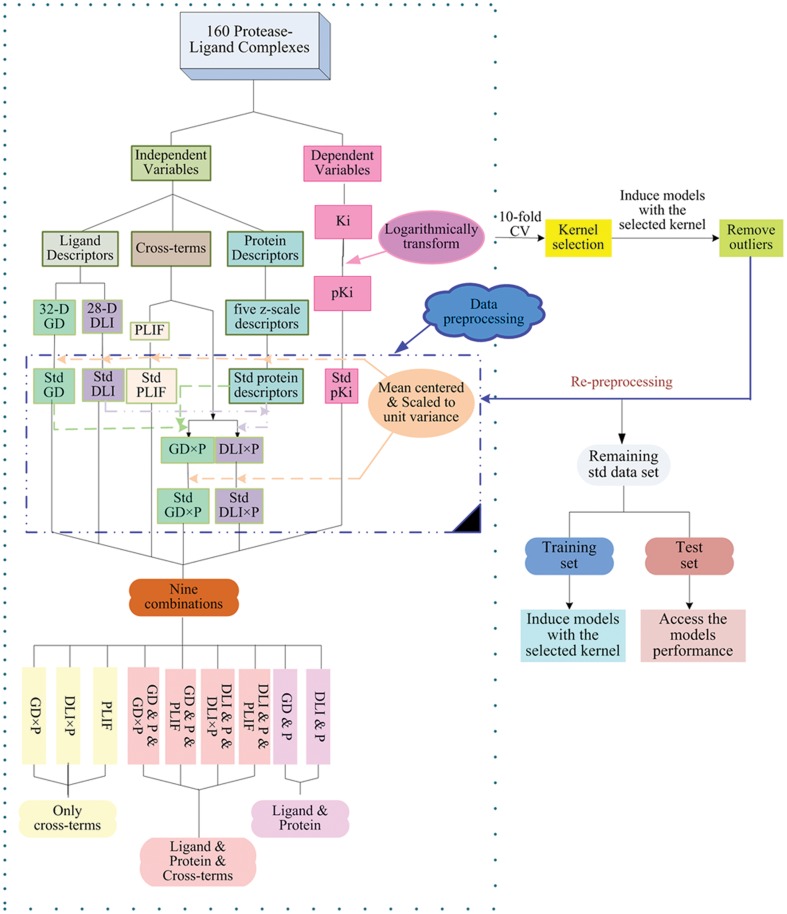
Graphical illustrations of the goodness-of-fit and predictive ability of the obtained models with the selected kernel. Goodness-of-fit is shown as red solid circles, and predictive ability is shown as blue solid circles. The predicted versus measured activity values using different combinations of descriptor blocks, i.e. GD×P (a), DLI×P (b), PLIF (c), GD & P & GD×P (d), GD & P & PLIF (e), GD & P (f), DLI & P & DLI×P (g), DLI & P & PLIF (h), DLI & P (i) are shown in the figure.

Diverse Subset method was used to split the remaining dataset into a training set (95 inhibitor-protease pairs) (see **Table S1**) and a test set (45 inhibitor-protease pairs) (see **Table S2**). The training set was used to create models and the test set was used to evaluate the performance of different models with different descriptor combinations. The obtained goodness-of-fit (R^2^) and predictive ability (Q^2^_test_) of models were illustrated in **Table 3** and **Figure 1**. As a result, nine new PCM models were obtained, and six of them achieved reasonably good predictive ability (Q^2^_test_>0.7). The results indicate that the SVR with the selected kernel, as well as the data partition strategy *etc* are all suitable for the present study.

**Table 3:** Goodness-of-fit (R^2^) and predictive ability (Q^2^_test_) of the obtained models.

Models with different descriptor combinations	GD		DLI	
	R^2^	Q^2^_test_	R^2^	Q^2^_test_
PLIF^a^	***0.9621***	***0.7470***	***0.9621***	***0.7470***
MLPD^a^	0.9700	0.7101	0.9722	0.6702
L & P & PLIF^b^	***0.9716***	***0.8271***	***0.9731***	***0.7929***
L & P &MLPD^b^	0.9696	0.7129	0.9727	0.6612
L&P^c^	0.9350	0.7298	0.9241	0.6134

a Models created using only cross-terms.

b Models created using ligand and protein descriptors with cross-terms.

c Models created using ligand and protein descriptors.

### Performance of PLIF as Cross-terms in Proteochemometric Modeling

From **Table 3**, we found that when including the PLIF cross-terms, the models obtained better predictive abilities than that of the models without PLIF. For the comparison purpose, we also used the conventional cross-terms MLPD to build PCM models, which is commonly used in previous proteochemometric modeling studies [22], [23], [24], [25], [26]. Obviously, for each kind of ligand descriptors, the newly introduced cross-terms PLIF outperformed the conventional MLPD whether we used only the cross-terms or the combinations of ligand, protein descriptors and cross-terms blocks to create models (see Table 3).

Cross-terms are influenced by both the ligand and the target part [31]. They are intended to describe the properties of the interface between ligand and protein. PLIF is a kind of interaction fingerprint which is calculated from the ligand-target complexes and directly describes the interaction of ligand with protein from hydrogen bonds, ionic interactions, and surface interactions [33]. Therefore, PLIF is inherent to be a suitable cross-term with no surprising that the model performance would be improved by using PLIF as cross-terms. In contrast, MLPD is derived by multiplying ligand and protein descriptors, which is not an essential reflection of the ligand-protein binding. In addition, our results also displayed that the use of MLPD as cross-terms could not improve the model performance significantly as PLIF did, and sometimes even deteriorate the predictive ability. Such result is probably explained by that the PCM models in this study were created using support vector machine, which is a non-linear machine learning method, which is actually expected to fulfill the same purpose as the MLPD does. Therefore, we may conclude that only when a suitable cross-term such as PLIF is used in proteochemometric modeling, the model performance can be improved significantly.

### Bioactivity Spectra of HIV-1 Protease Inhibitors

**Figure 2:**
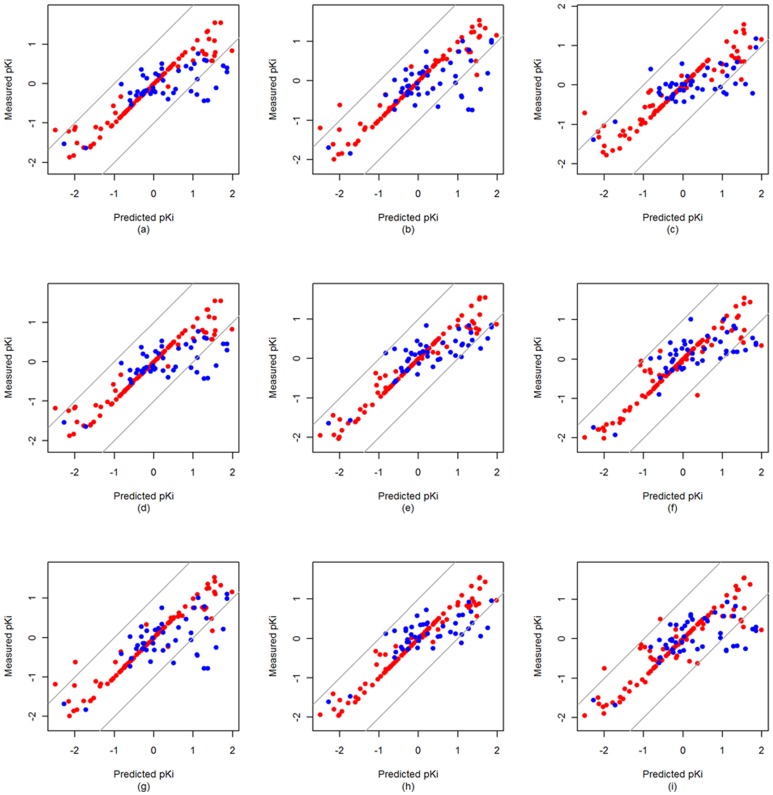
Predicted inhibitory activity (pKi) of the selected eight compounds against 47 proteases. Red, pink, brown, orange circles stand for the first-generation inhibitors, i.e. Saquinavir, Ritonavir, Indinavir, Nelfinavir respectively; Darkgreen, cadetblue, cyan, blue triangles stand for the second-generation ones, i.e. Darunavir, Tipranavir, TMC-126, XV638 respectively. The lines indicate the average values for each of them.

Bioactivities of the four first-generation and four second-generation inhibitors against the 47 protease variants were predicted using our selected best PCM model. The results (shown in **Figure 2**) display that the predicted activities of the second-generation inhibitors are higher than the first-generation ones for most variants. The average predicted values of the second-generation ones are also higher than that of the first-generation ones. Furthermore, the number of proteins for the eight inhibitors whose predicted activities are higher than zero is 10 for Saquinavir, 15 for Ritonavir, 15 for Indinavir, 12 for Nelfinavir, 22 for Darunavir, 23 for Tipranavir, 21 for TMC-126 and 25 for XV638 respectively.

As we all know, the arrival of the early HIV-1 protease inhibitors was a pivotal moment in the development of antiretroviral therapy. However, the rapid emerging resistance to the first-generation of protease inhibitors occurred, which brought a substantial and persistent problem in the treatment of AIDS. Hence, to inhibit these drug-resistant HIV protease variants, second-generation approaches have been developed. As a result, the second-generation inhibitors should have a broader antiviral activity. Meanwhile, all the above-mentioned results also indicate that the second-generation inhibitors are potent against a wider spectrum of protease variants. Thus it can be seen that our derived model provides a useful way to discovery novel inhibitors which have a broad antiviral activity.

### Conclusions

To sum up, we have successfully applied proteochemometric modeling in the study of the bioactivity spectra of HIV-1 protease inhibitors and introduced a new cross-term PLIF into proteochemometrics. Our results showed that when cross-terms were introduced into proteochemometric modeling, the newly introduced cross-term PLIF could always improve the model performance significantly. In addition, we also found that PLIF had a better predictive ability than that of the conventional MLPD. Furthermore, our best derived model shows the ability to discover novel inhibitors with broad antiviral activity. Our study indicates that PLIF could improve the resolution and predictive ability of the PCM model and consequently have potential application to solve the HIV-1 drug-resistant problem.

## Materials and Methods

### Data set

To create PCM models with PLIF, protein-ligand complexes of HIV-1 protease variants with their inhibitors and the corresponding activity values were collected. Activity is described with *K*_i_ value which is an inhibition constant and less susceptible by the experiment circumstances than the others such as IC_50_, EC_50_ and *etc*. As a result, 160 protease-ligand complexes with inhibition constants (*K*_i_) were retrieved from PDB database, including 92 chemical compounds and 47 HIV-1 protease variants. Inhibition constants of the 160 unique inhibitor-protease pairs were collected from the literatures (see **Table S1, S2 and S3**). More recent studies suggest that not only the active-site mutations but also distant mutations may influence the drugs' ability to inhibit the protease [41], thus all the mutations in the variants should be taken into consideration in the design of the inhibitors. The fragment 501–599 of P03366 protein was considered as a wild-type protease, and all of the proteases in the 160 complexes were aligned to the wild-type. As a result, the number of the sequences of mutated proteases differing from the wild-type sequence ranges from one to twenty-one (5.17 on average). In all the data set, 69 compounds are collected with *K*_i_ value for only one type of HIV-1 protease variant, and there are 22 protease variants which only have one ligand with collected *K*_i_ value for each of them.

**Figure 3:**
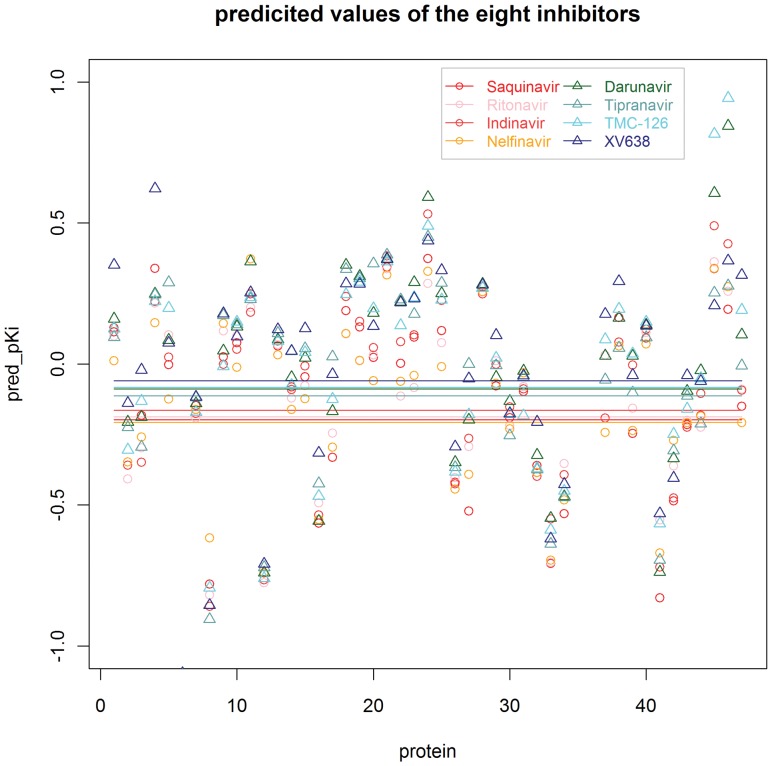
General framework for our proteochemometric modeling.

The data set was divided into a training set (67%) and an external test set (33%) according to the Diverse Subset partition strategy built in MOE [33], which is used to rank entries in a database based on the distance of their sequences from each other. The general framework for our proteochemometric modeling is presented in **Figure 3**.

### Numerical Descriptions for Proteochemometric Modeling

#### Description of Proteases

Of the 99 amino acids in each protease monomer, 48 positions were found to be mutated in the data set. Mutated positions were coded using the five z-scale descriptors, z1–z5, of amino acids derived by Sandberg et al [42]. The five z-scales are the principal components of 26 computed and measured physicochemical properties of amino acids, and represent essential hydrophobicity/hydrophilicity (z1), steric bulk properties and polarizability (z2), polarity (z3), and electronic effects (z4 and z5) of amino acids. In this way, the varying parts of protease sequences were represented by 48×5 = 240 protease descriptors.

#### Description of Protease Inhibitors

The inhibitors were represented with two typical kinds of feature space respectively, i.e. 32-dimensional general descriptors (GD) and 28-dimensional Drug-Like Index (DLI). The two sets of descriptors are widely applied in describing organic compounds, and they describe compounds from the views of intrinsic characteristics and drug-like properties respectively. GD includes atomic contributions to logP, molar refractivity, and atomic partial charge [43]. These descriptors characterize physical properties of compounds. They were successfully used to build reasonably good QSAR/QSPR models of boiling point, vapor pressure, free energy of salvation in water, water solubility, receptor class, activity against thrombin/trypsin/factor Xa, blood-brain barrier permeability and compound classification etc. On the other hand, DLI characterizes the hierarchy of drug structures in terms of rings, links, and molecular frameworks [44]. DLI was initially used to rank compounds in a library to select drug-like compounds. In contrast to GD, DLI characterizes simple topological indices of compounds. Therefore, the obtained models will be validated with the two sets of features respectively.

#### Protease-Inhibitor Cross-terms

Interaction fingerprints have been developed to enhance the representation and analysis of three-dimensional protein-ligand interactions, such as SIFt (structure interaction fingerprint) [45], APIF (atom-pairs-based interaction fingerprint) [46], Pharm-IF (pharmacophore-based interaction fingerprint) [32], PLIF (protein-ligand interaction fingerprint) [32], [33] etc. Additionally, MM-PBSA/GBSA [47], [48], [49], [50] can also generate protein-ligand interaction spectra, which is based on the binding energy. Here, protein-ligand interaction fingerprints were calculated as protease-inhibitor cross-terms using the functions built in MOE [33]. PLIF summarizes the interactions between ligands and proteins using a fingerprint scheme. The interactions are classified into six types: sidechain hydrogen bonds (donor or acceptor), backbone hydrogen bonds (donor or acceptor), ionic interactions, and surface interactions in which a residue may participate. Setting the “Maximum # Bits” as 1000, and using the other default settings, raw protein-ligand interaction data were calculated, and then fingerprint bits were generated. Finally, totally 46 descriptors were extracted whose values represented the strength of the corresponding interaction with the ligand (these 46 descriptors were listed in **Table S4**). In order to assess the efficiency of introduction of PLIF, MLPD (GD: 32×240 = 7680 cross-terms or DLI: 28×240 = 6720 cross-terms) was also adopted for a complementary comparison.

### Preprocessing of data

Prior to the calculation of MLPD and further building PCM models, all descriptors were mean centered and scaled to unit variance. The dependent variable (*K*_i_) was logarithmically transformed and also mean centered and scaled to unit variance prior to the use in the computations..

### Proteochemometrics Modeling

#### Selection of Kernel of Support Vector Regression

All models were created using support vector regression (SVR) built in the Weka [36] suit (Weka implementation “SMOreg”), which is a collection of machine learning algorithms for data mining tasks. The kernel of SMOreg implemented in Weka consists of Normalized Poly Kernel (normalized polynomial kernel), Poly Kernel (polynomial kernel), Precomputed Kernel Matrix Kernel, Puk (Pearson VII function-based universal kernel), RBF Kernel (Radial Basis Function kernel), String Kernel. Since Precomputed Kernel Matrix Kernel is based on a static kernel matrix that is read from a file, and String Kernel can't handle multi-valued nominal attributes, the kernel was selected from the left four nonlinear functions. The SMOreg algorithm was run with no normalization/standardization on each of the four kernels. The efficacy of the four kernels was assessed by Q^2^ (predictive ability) with 10-fold cross-validation.

#### Model Induction and Validation

We used nine different combinations of descriptor blocks, i.e. three kinds of cross-terms (PLIF, MLPD of GD×P, MLDP of DLI×P), two combinations of ligand and protein descriptors without cross-term (GD & P, DLI & P), and four combinations of ligand, protein descriptors with cross-terms (GD & P & PLIF, GP & P & GP×P, DLI & P & PLIF, DLI & P& DLI×P) to create models from all the datasets with the selected kernel. The Z score method was adopted for the detection of outliers [51], [52], [53]. Any pair is considered as an outlier with removing, if it shows a value of Z-score no lower than 2.0 in no less than five of these nine models. Then the left datasets were split into a training set and a test set. We created nine new models with the training set and assessed the models performance with the external test set. At last, the derived models were quantified by the goodness-of-fit (R^2^) and predictive ability (Q^2^_test_).

Finally, we selected four first-generation inhibitors, which are the first four drugs (Saquinavir, Ritonavir, Indinavir and Nelfinavir) [54] approved by Food and Drug Administration (FDA), and four second-generation ones, of which two (Darunavir [55] and Tipranavir [56]) are the most recently approved drugs [54] and the other two (TMC-126 [57] and XV638 [58]) are reported to be extremely potent against a wide spectrum of HIV. If there were no experimental complexes for the eight inhibitors against the 47 protease variants, these inhibitors were docked into the protease to derive complexes and generate PLIFs using MOE (presented in **Table S5**). Subsequently, the best derived model was used to predict their bioactivity spectra.

## Supporting Information

Table S1 **Training set used for construction of the proteochemometric models.**
(DOCX)

Table S2 **Test set used for assessment of the proteochemometric models.**
(DOCX)

Table S3 **Removed outliers.**
(DOCX)

Table S4 **PLIFs for all the experimental complexes.**
(XLSX)

Table S5 **PLIFs for the eight inhibitors against all the proteases.**
(XLSX)
