# Supplementary material for: Proteochemometric Modeling of the Bioactivity Spectra of HIV-1 Protease Inhibitors by Introducing Protein-Ligand Interaction Fingerprint
Source: PLoS One. 2012 Jul 27;7(7):e41698. doi: 10.1371/journal.pone.0041698 (PMC3407198; doi:10.1371/journal.pone.0041698)
Supplement: Table S1 — Training set used for construction of the proteochemometric models. (DOCX) [file pone.0041698.s001.docx]

**Table S1.** Training set

| **Protease number** | **Mutation** | **Ligand** | **PDBid** | **Ki(nM)** | **Ref** |
| --- | --- | --- | --- | --- | --- |
| 1 | I3V, V82F | DMP323  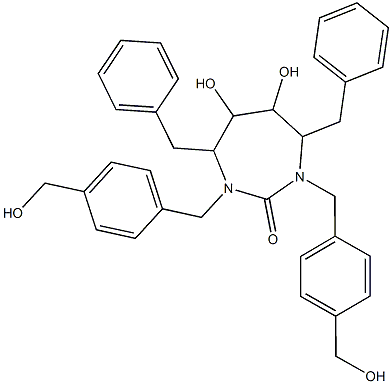 | 1MET | 0.4 | [[1](#_ENREF_1)] |
| 1 | I3V, V82F | XV638  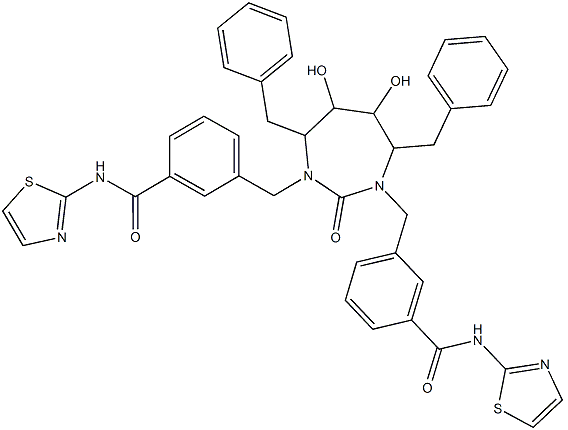 | 1BV7 | 0.5 | [[2](#_ENREF_2)] |
| 2 | I3V, V82F, I84V | XV638  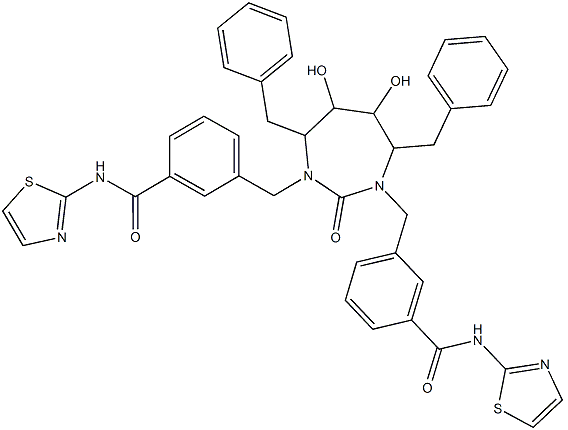 | 1BWA | 25 | [[2](#_ENREF_2)] |
| 2 | I3V, V82F, I84V | SD146  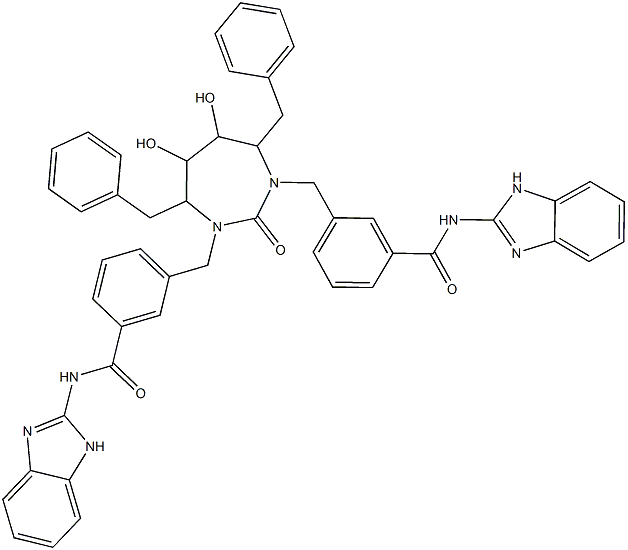 | 1BWB | 38 | [[2](#_ENREF_2)] |
| 3 | I3V, I84V | XV638  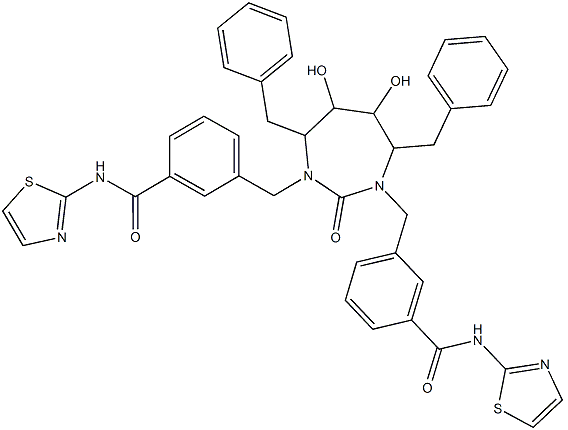 | 1BV9 | 1.1 | [[2](#_ENREF_2)] |
| 3 | I3V, I84V | DMP450  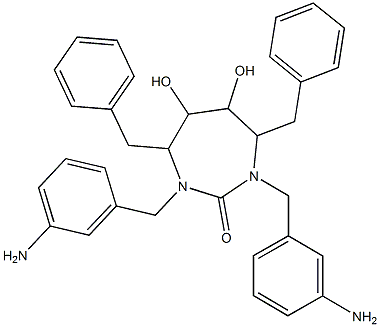 | 1MER | 10 | [[1](#_ENREF_1)] |
| 3 | I3V, I84V | DMP323  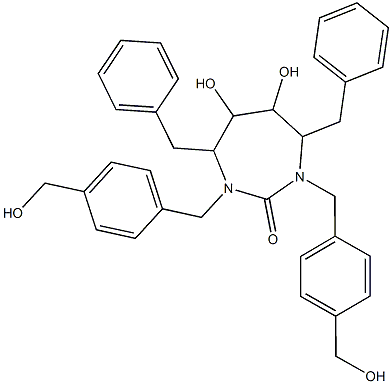 | 1MES | 20 | [[1](#_ENREF_1)] |
| 4 | I3V, C95A | XK216  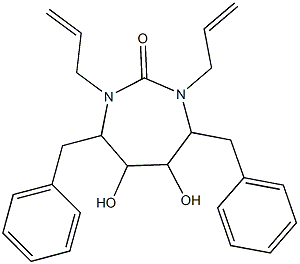 | 1HWR | 4.7 | [[3](#_ENREF_3)] |
| 4 | I3V, C95A | SD146  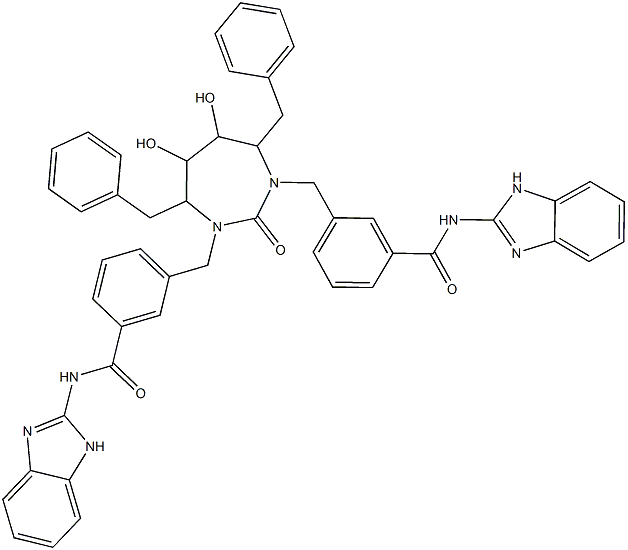 | 1QBT | 0.024 | [[4](#_ENREF_4)] |
| 4 | I3V, C95A | XV638  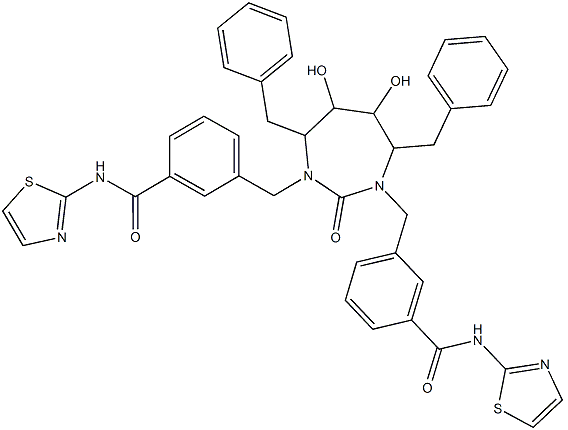 | 1QBR | 0.027 | [[4](#_ENREF_4)] |
| 4 | I3V, C95A | DMP450  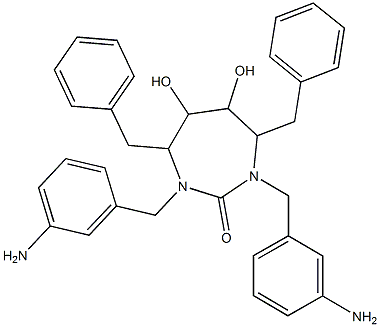 | 1DMP | 0.28 | [[5](#_ENREF_5)] |
| 5 | Q7K | 3TL  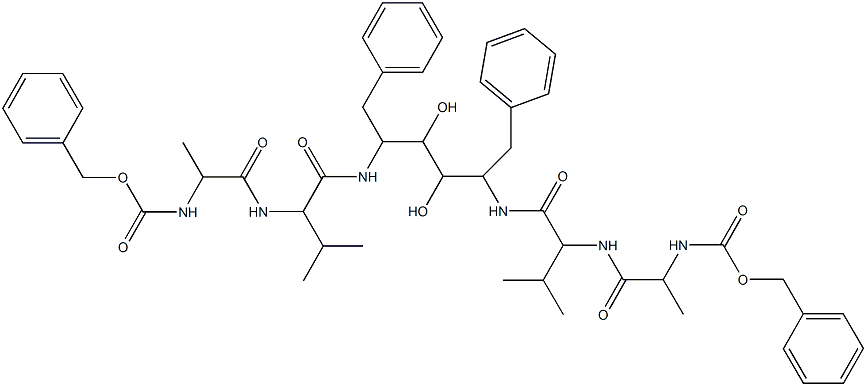 | 2P3B | 3.3 | [[6](#_ENREF_6)] |
| 5 | Q7K | Lopinavir  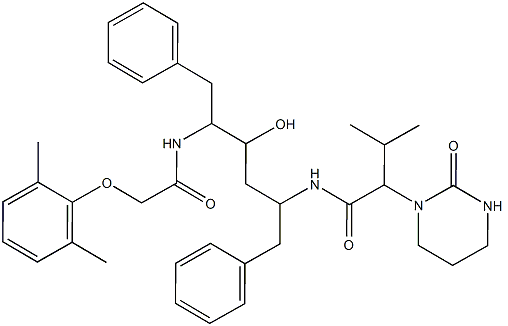 | 2O4S | 0.031 | [[7](#_ENREF_7)] |
| 6 | Q7K, L10I, I15V, G16E, K20R, E35D, M36I, S37N, P39S, R41K, M46I, G51R, I54V, R57K, D60E, Q61D, K70R, I72V, T74A, V82A, L89M | 3TL  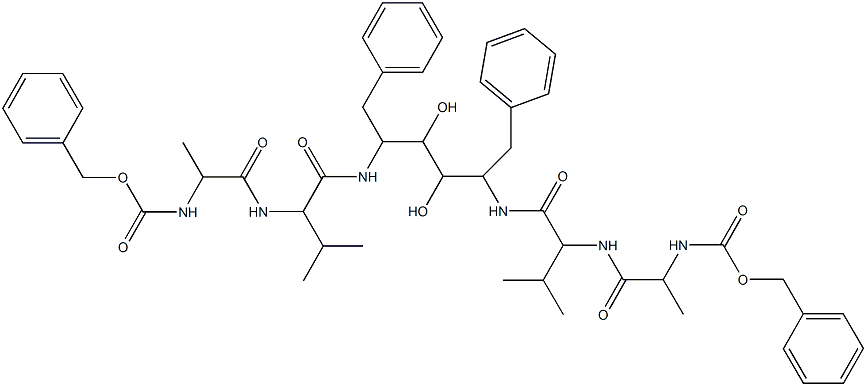 | 2P3D | 180 | [[6](#_ENREF_6)] |
| 7 | Q7K, T12S, I15V, L33I, M36I, S37A, L63I, H69K, L89M | Indinavir  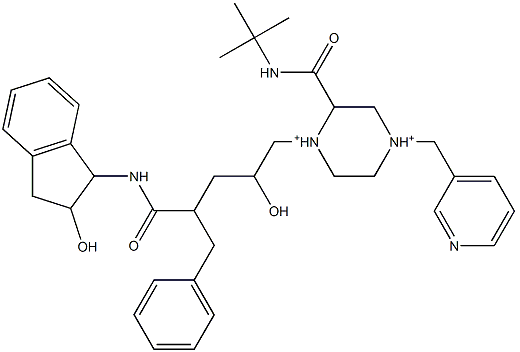 | 2R5P | 3.3 | [[8](#_ENREF_8)] |
| 7 | Q7K, T12S, I15V, L33I, M36I, S37A, L63I, H69K, L89M | Nelfinavir  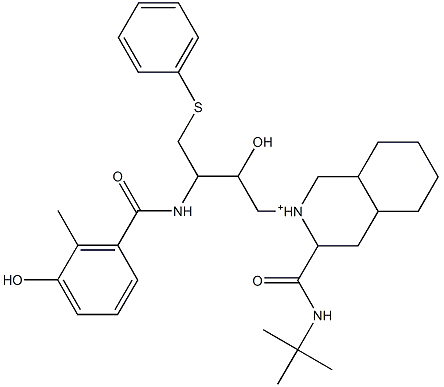 | 2R5Q | 2.7 | [[8](#_ENREF_8)] |
| 8 | Q7K, I13V, V32I, L33F, K45I, V82L, I84V | Tipranavir  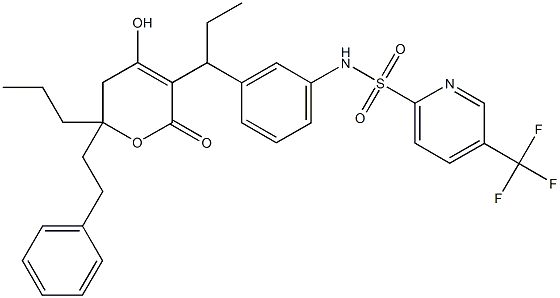 | 2O4N | 27.65 | [[7](#_ENREF_7)] |
| 8 | Q7K，K14R, L33I，S37N, R41K, L63P, I64V, C67Aba，C95Aba | HBB  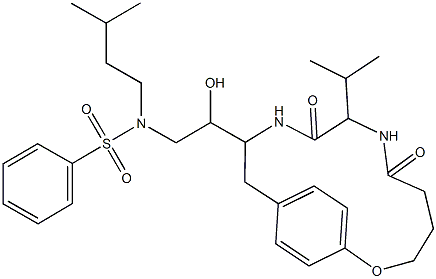 | 1Z1H | 4 | [[9](#_ENREF_9)] |
| 8 | Q7K，K14R, L33I，S37N, R41K, L63P, I64V, C67Aba，C95Aba | PI4  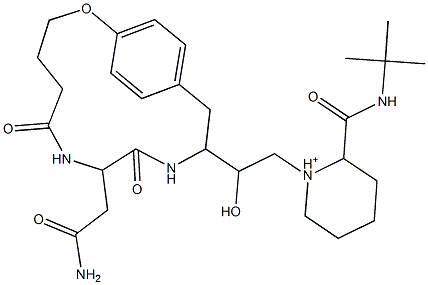 | 1B6L | 5 | [[9](#_ENREF_9)] |
| 10 | Q7K, K14R, S37N, R41K, L63P | K60  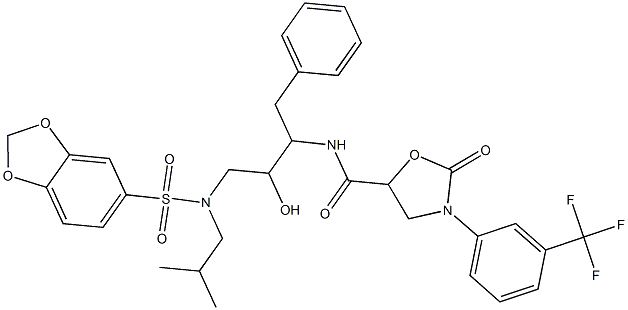 | 3GI4 | 0.016 | [[10](#_ENREF_10)] |
| 10 | Q7K, K14R, S37N, R41K, L63P | MU1  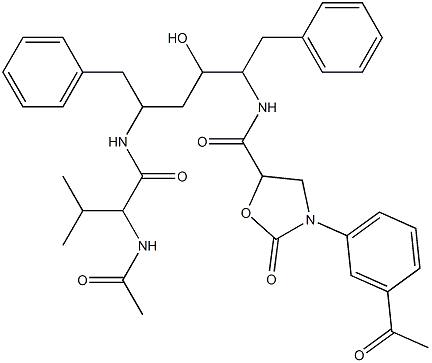 | 2Q54 | 0.98 | [[11](#_ENREF_11)] |
| 10 | Q7K, K14R, S37N, R41K, L63P | MZ2  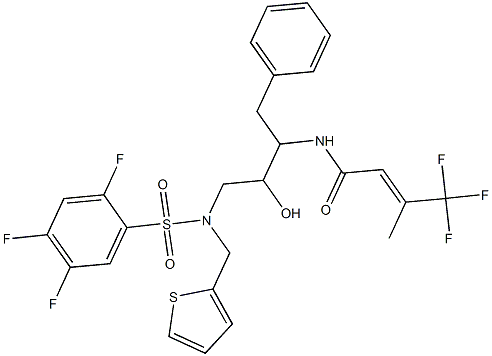 | 2QHZ | 53 | [[12](#_ENREF_12)] |
| 10 | Q7K, K14R, S37N, R41K, L63P | MZ4  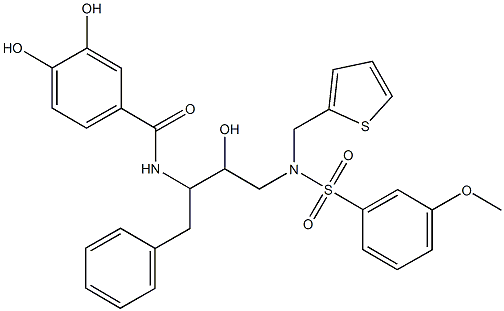 | 2QI1 | 50 | [[12](#_ENREF_12)] |
| 10 | Q7K, K14R, S37N, R41K, L63P | MZ5  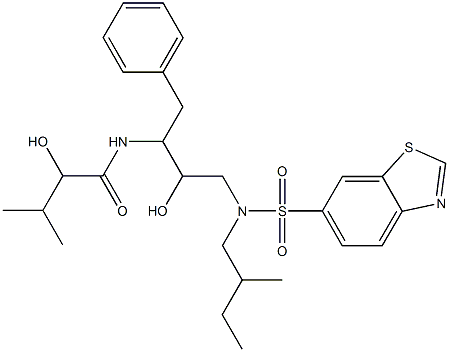 | 2QI3 | 0.063 | [[12](#_ENREF_12)] |
| 11 | Q7K, K14R, S37N, R41K, L63P, I64V | KNI-764  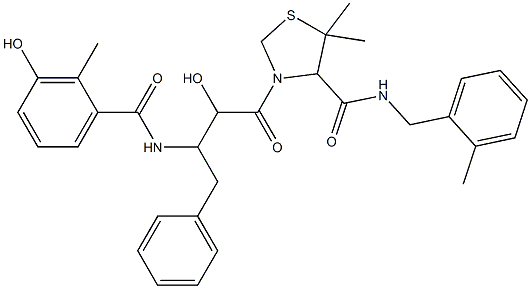 | 1KZK | 0.041 | [[13](#_ENREF_13)] |
| 12 | Q7K, I15V, E35D, M36I, S37A, R41K, R57K, D60E, Q61N, I62V, L63S, I64L, L89M | 3TL  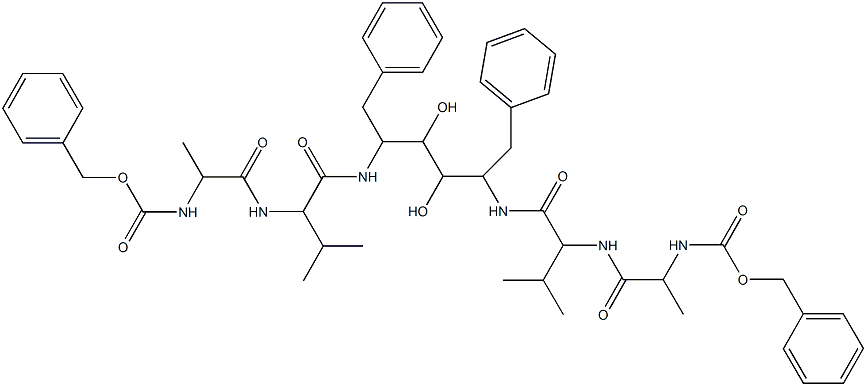 | 2P3C | 24 | [[6](#_ENREF_6)] |
| 13 | Q7K, L24I, L33I, L63I, C67A, C95A | Indinavir  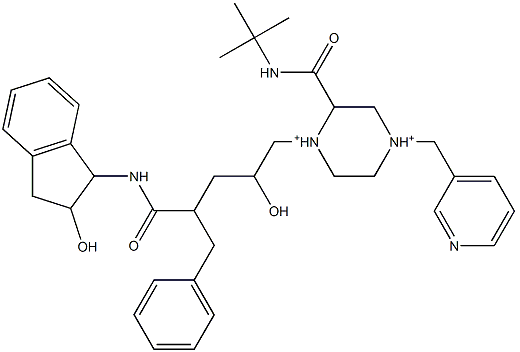 | 2AVO | 1.4 | [[14](#_ENREF_14)] |
| 14 | Q7K, D30N, L33I, L63I, C67A, C95A | 065  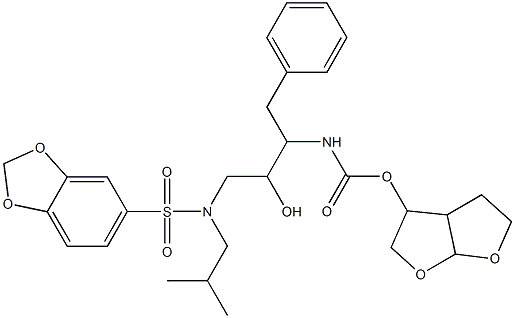 | 2QCI | 4.6 | [[15](#_ENREF_15)] |
| 15 | Q7K, V32I, L33I, L63I, C67A, C95A | Amprenavir  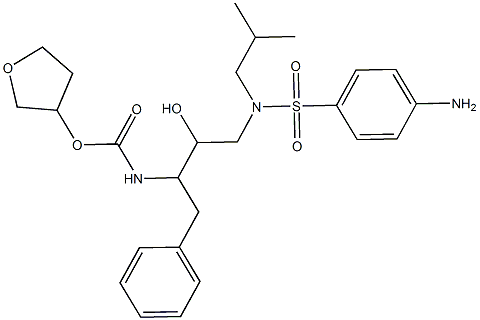 | 3NU4 | 1.5 | [[16](#_ENREF_16)] |
| 16 | Q7K, L33I, G48V, L63I, C67A, C95A | Darunavir  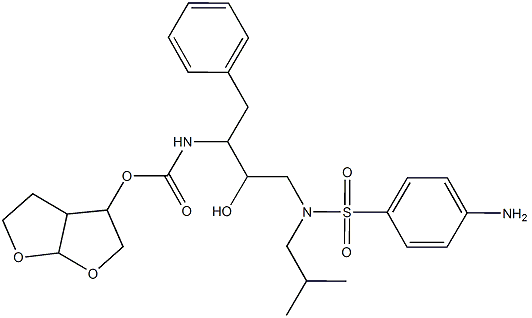 | 3CYW | 17 | [[17](#_ENREF_17)] |
| 17 | Q7K, L33I, I50V, L63I, C67A, C95A | 065  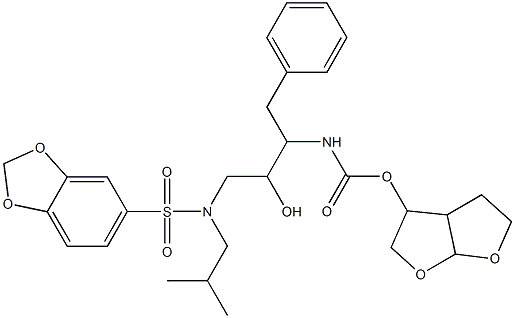 | 2QD6 | 2.1 | [[15](#_ENREF_15)] |
| 17 | Q7K, L33I, I50V, L63I, C67A, C95A | Saquinavir  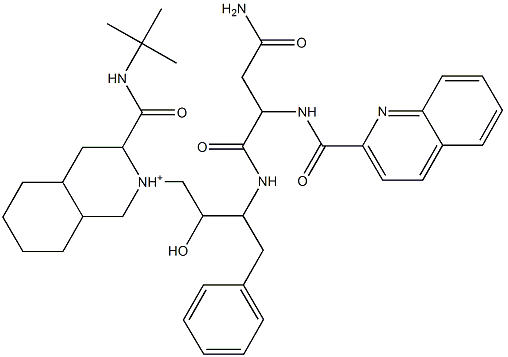 | 3CYX | 10 | [[17](#_ENREF_17)] |
| 17 | Q7K, L33I, I50V, L63I, C67A, C95A | Indinavir  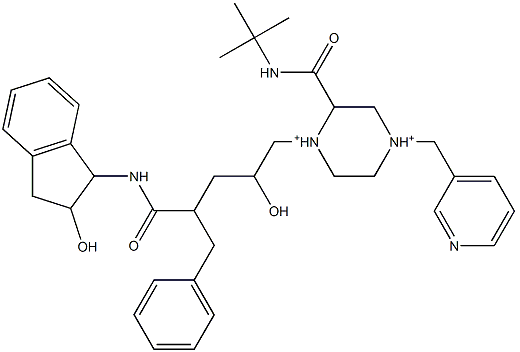 | 2AVS | 24 | [[14](#_ENREF_14)] |
| 18 | Q7K, L33I, I54M, L63I, C67A, C95A | Amprenavir  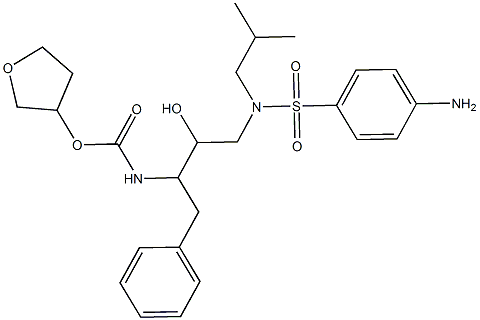 | 3NU6 | 0.5 | [[16](#_ENREF_16)] |
| 18 | Q7K, L33I, I54M, L63I, C67A, C95A | Saquinavir  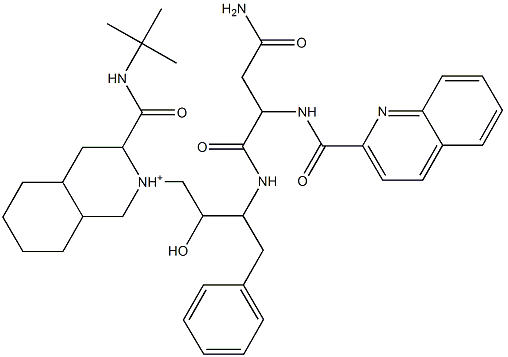 | 3D1X | 2.2 | [[17](#_ENREF_17)] |
| 19 | Q7K, L33I, I54V, L63I, C67A, C95A | Amprenavir  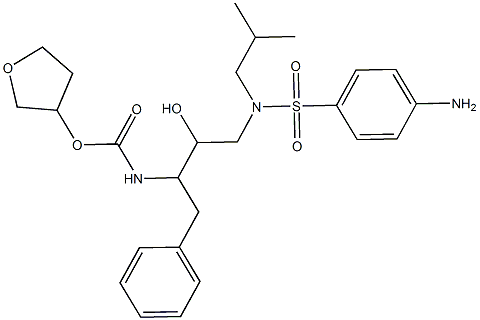 | 3NUJ | 0.41 | [[16](#_ENREF_16)] |
| 19 | Q7K, L33I, I54V, L63I, C67A, C95A | Saquinavir  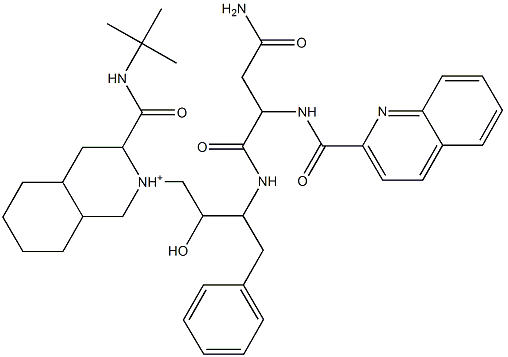 | 3D1Y | 6 | [[17](#_ENREF_17)] |
| 20 | Q7K, L33I, L63I | Tipranavir  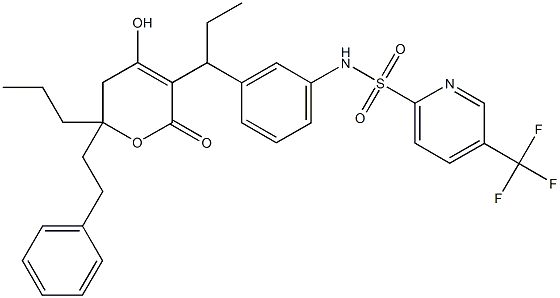 | 1D4Y | 0.008 | [[18](#_ENREF_18)] |
| 20 | Q7K, L33I, L63I | UNI  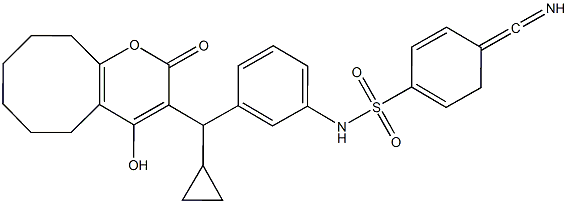 | 1HPO | 0.6 | [[19](#_ENREF_19)] |
| 21 | Q7K, L33I, L63I, C67A, G73S, C95A | Indinavir  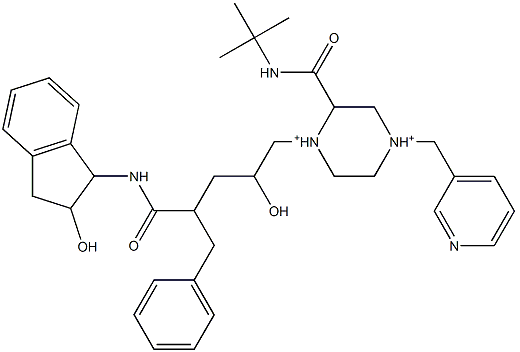 | 2AVV | 0.55 | [[14](#_ENREF_14)] |
| 22 | Q7K, L33I, L63I, C67A, V82A, C95A | Saquinavir  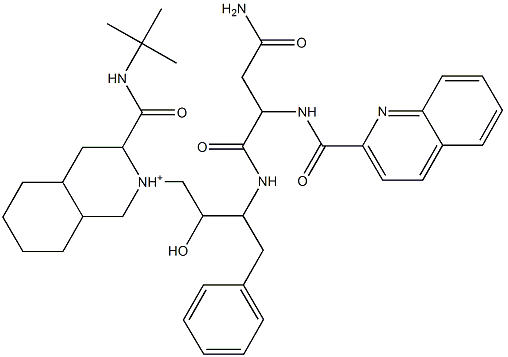 | 2NMZ | 4.3 | [[20](#_ENREF_20)] |
| 22 | Q7K, L33I, L63I, C67A, V82A, C95A | 065  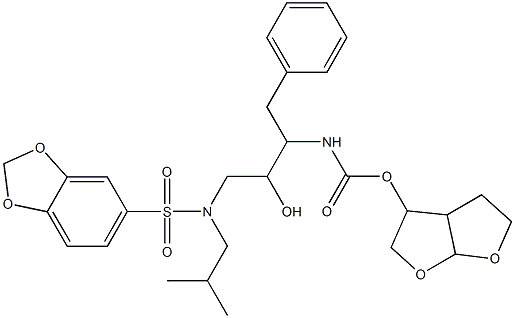 | 2QD7 | 0.8 | [[15](#_ENREF_15)] |
| 22 | Q7K, L33I, L63I, C67A, V82A, C95A | Indinavir  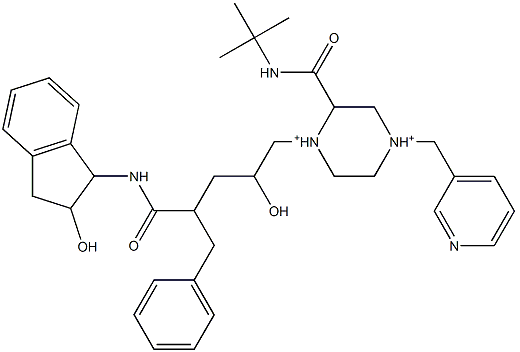 | 1SDV | 1.81 | [[21](#_ENREF_21)] |
| 23 | Q7K, L33I, L63I, C67A, I84V, C95A | 065  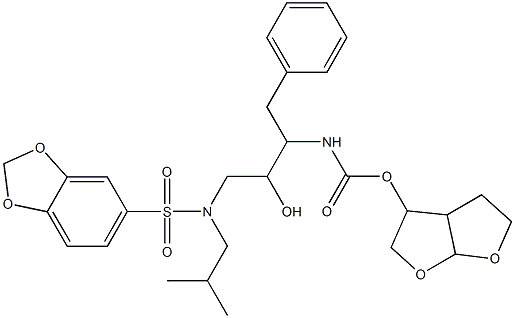 | 2QD8 | 0.85 | [[15](#_ENREF_15)] |
| 23 | Q7K, L33I, L63I, C67A, I84V, C95A | Saquinavir  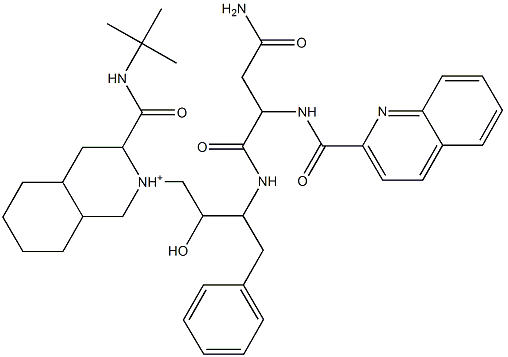 | 2NNK | 4.3 | [[20](#_ENREF_20)] |
| 24 | Q7K, L33I, L63I, C67A, L90M, C95A | Amprenavir  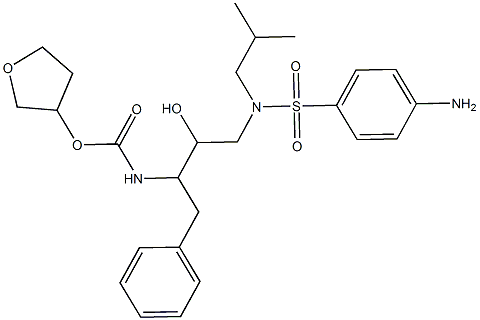 | 3NUO | 0.16 | [[16](#_ENREF_16)] |
| 24 | Q7K, L33I, L63I, C67A, L90M, C95A | Indinavir  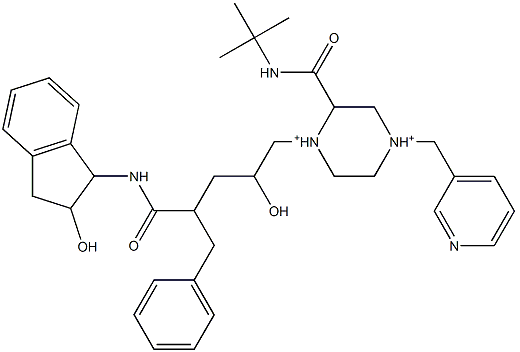 | 1SDU | 0.086 | [[21](#_ENREF_21)] |
| 25 | Q7K, L33I, L63I, C67A, C95A | Indinavir  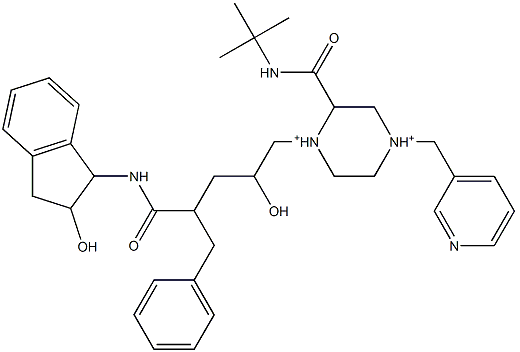 | 1SDT | 0.54 | [[21](#_ENREF_21)] |
| 25 | Q7K, L33I, L63I, C67A, C95A | Darunavir  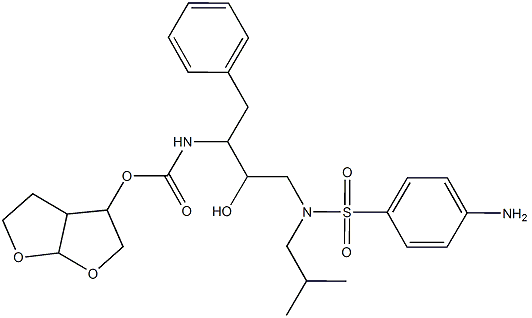 | 2IEN | 1 | [[22](#_ENREF_22)] |
| 25 | Q7K, L33I, L63I, C67A, C95A | Saquinavir  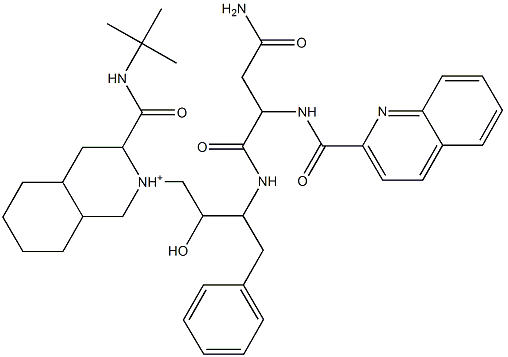 | 2NMW | 3.9 | [[20](#_ENREF_20)] |
| 26 | Q7K, S37A, R41K, K45R, I54V, L63P, A71V, V82A, L90M | 3TL  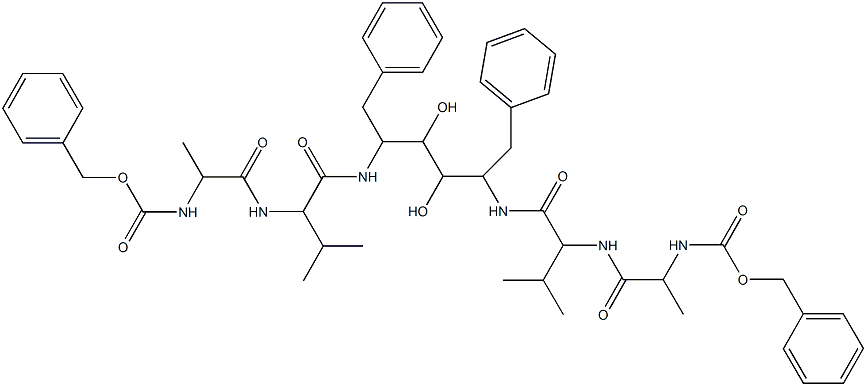 | 2P3A | 11 | [[6](#_ENREF_6)] |
| 27 | Q7K, I50V | Tipranavir  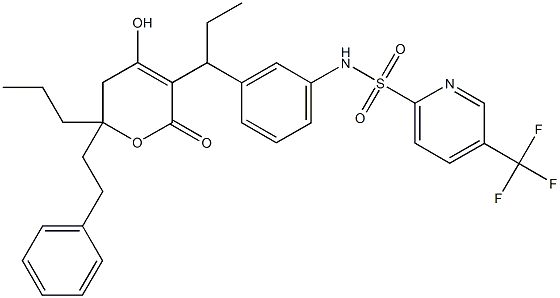 | 2O4L | 1.12 | [[7](#_ENREF_7)] |
| 28 | L10F, I13V, G16A, K20M, V32I, K43T, M46V, I47V, I54M, I64V, A71V, V82A | Lopinavir  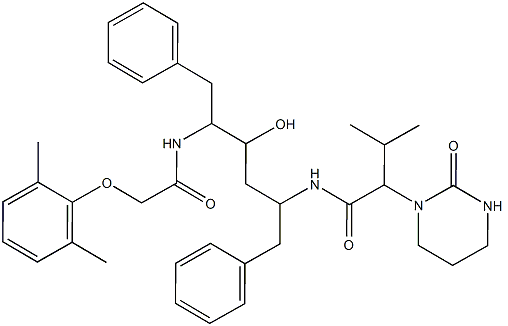 | 2RKF | 0.83 | [[23](#_ENREF_23)] |
| 29 | K14R, S37N, R41K, L63P, I64V, C67Aba，C95Aba | PI1  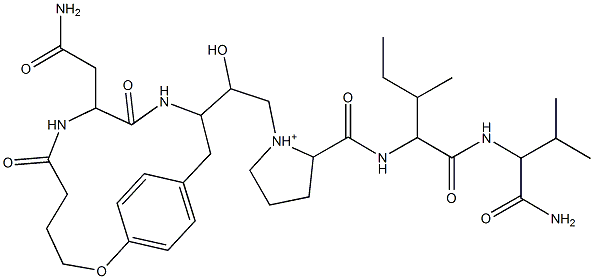 | 1B6J | 12 | [[9](#_ENREF_9)] |
| 31 | D30N, R41A | Nelfinavir  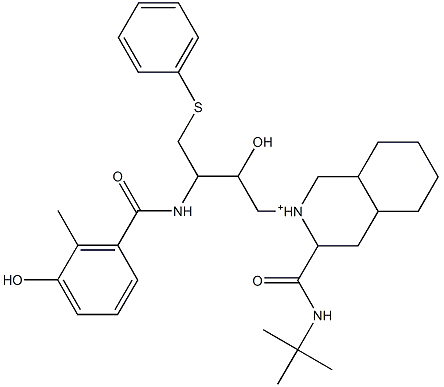 | 2Q64 | 1.4 | [[24](#_ENREF_24)] |
| 32 | D30N, R41A, A71V | Nelfinavir  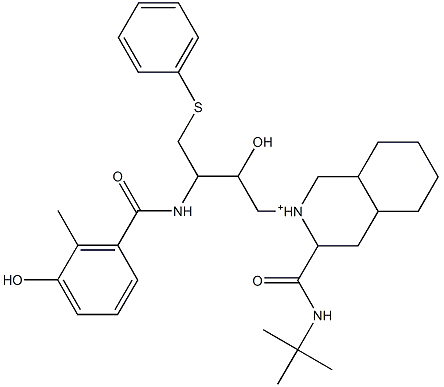 | 2PYN | 7 | [[24](#_ENREF_24)] |
| 33 | D30N, R41A, N88D | Nelfinavir  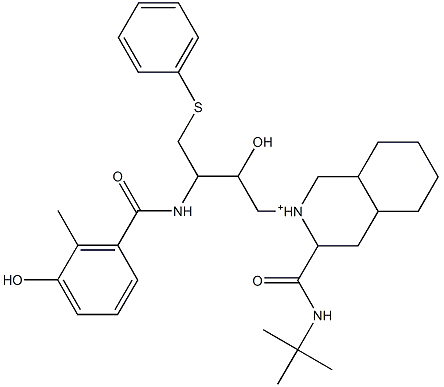 | 2PYM | 18 | [[24](#_ENREF_24)] |
| 34 | D30N, K55A, L90M | Nelfinavir  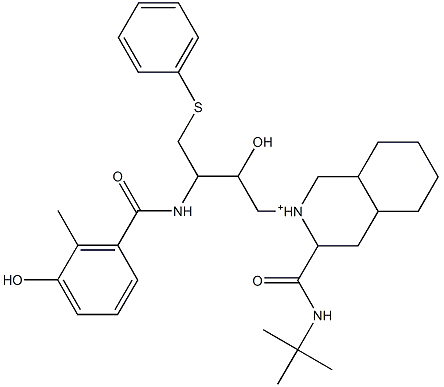 | 2Q63 | 9.9 | [[24](#_ENREF_24)] |
| 35 | T31S, V32I, L33V, E34A, E35G, M36I, S37E, I47V, V82I | SB 203386  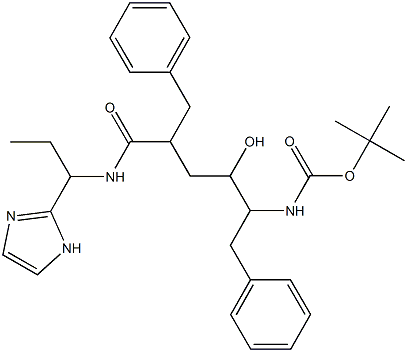 | 1BDQ | 460 | [[25](#_ENREF_25)] |
| 36 | T31S, L33V, E34A, E35G, M36I, S37E | SB 203386  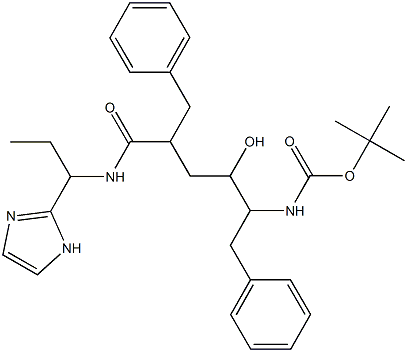 | 1BDR | 210 | [[25](#_ENREF_25)] |
| 37 | S37N | BEB  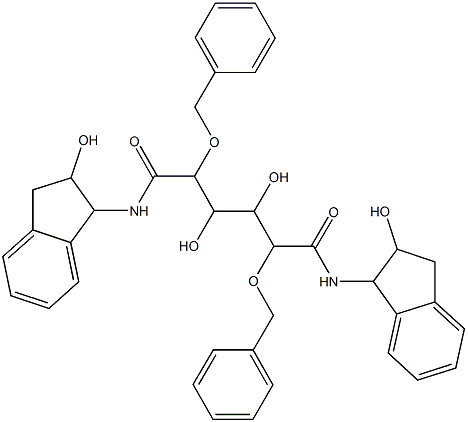 | 1EBY | 0.2 | [[26](#_ENREF_26)] |
| 37 | S37N | Ritonavir  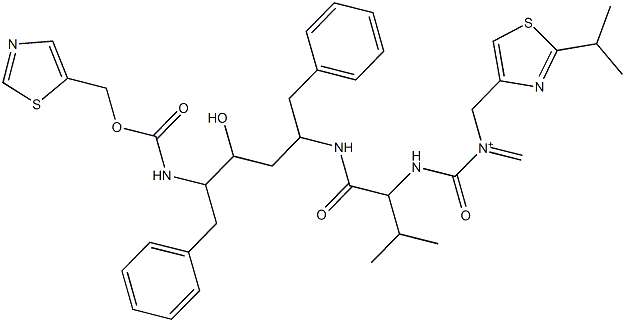 | 1HXW | 0.015 | [[27](#_ENREF_27)] |
| 37 | S37N | IM1  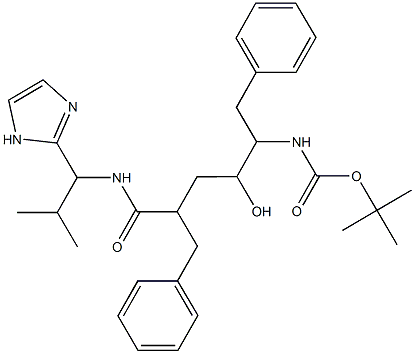 | 1SBG | 18 | [[28](#_ENREF_28)] |
| 38 | S37N, A71V, V82T, I84V | Q50  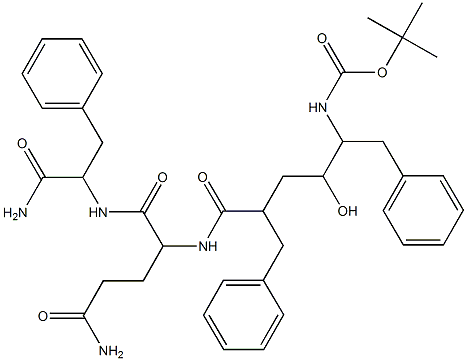 | 1IZI | 0.26 | [[29](#_ENREF_29)] |
| 39 | R41K, V82A | AI  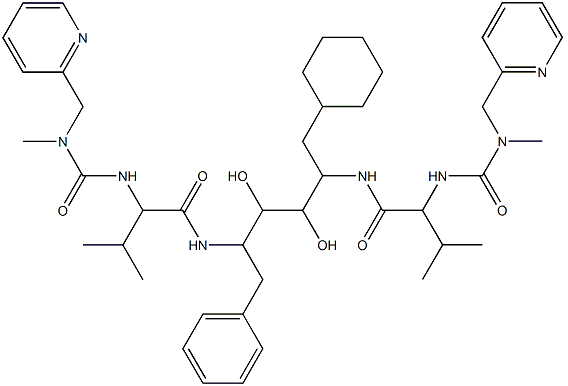 | 2FLE | 14 | [[30](#_ENREF_30)] |
| 40 | I47A | Lopinavir  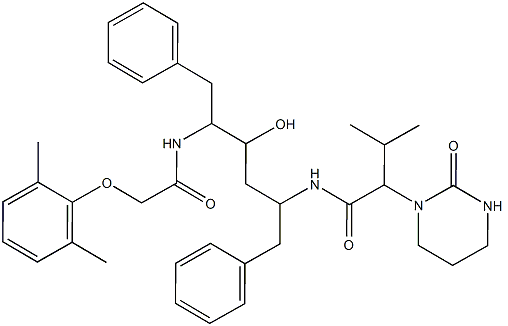 | 2QHC | 1.1 | [[31](#_ENREF_31)] |
| 41 | G48H | U-89360E  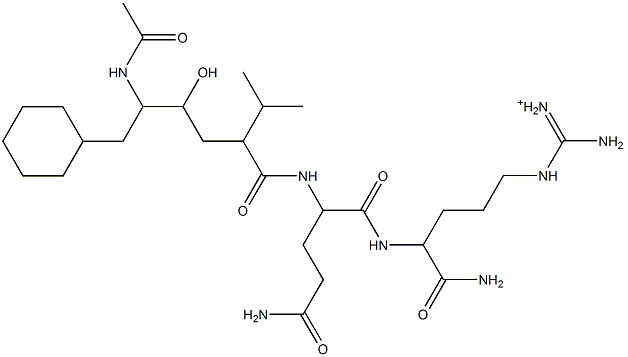 | 1A9M | 119 | [[32](#_ENREF_32)] |
| 42 | I50V | G4G  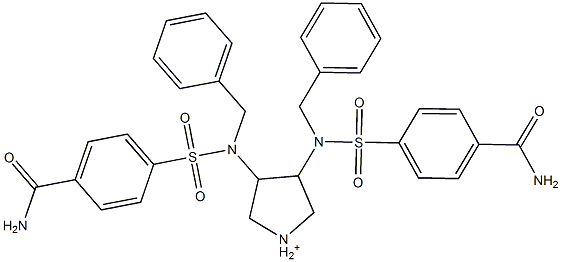 | 2R3T | 1400 | [[33](#_ENREF_33)] |
| 42 | I50V | G3G  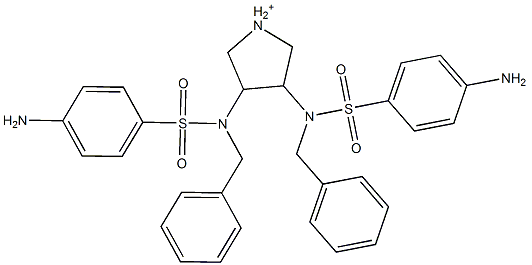 | 2R43 | 1000 | [[33](#_ENREF_33)] |
| 43 | L63P,V82T,I84V | 5AH  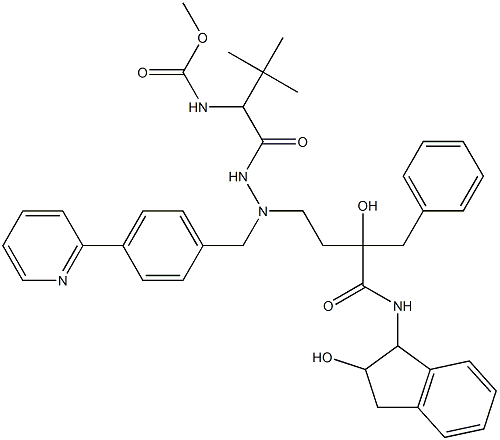 | 2WL0 | 16 | [[34](#_ENREF_34)] |
| 44 | I84V | G4G  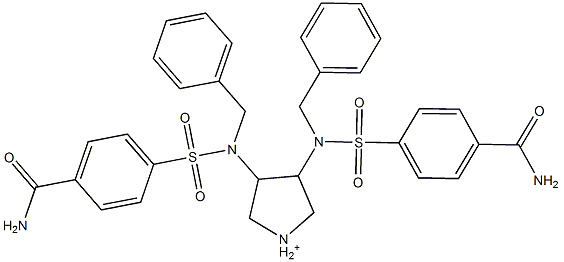 | 2R38 | 36 | [[33](#_ENREF_33)] |
| 44 | I84V | G3G  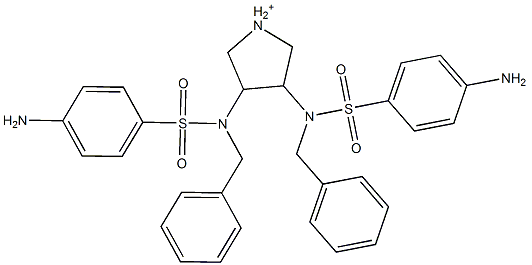 | 2R3W | 128 | [[33](#_ENREF_33)] |
| 45 | I84V, L90M, C95M | GS-8374  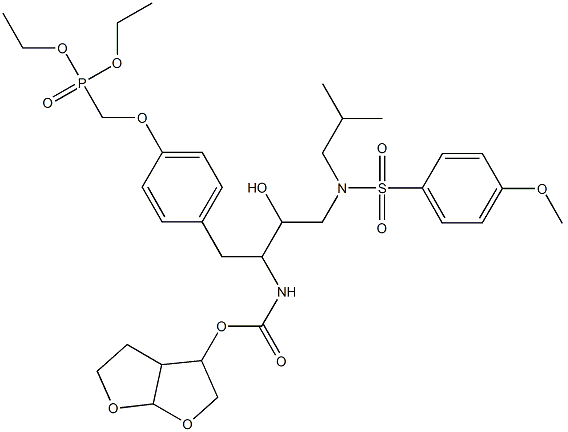 | 2I4X | 0.02888 | [[35](#_ENREF_35)] |
| 45 | I84V, L90M, C95M | TMC-126  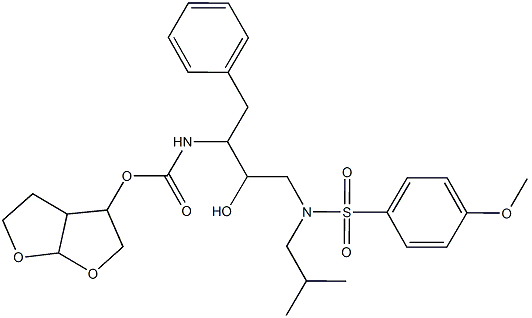 | 2I4V | 0.06111 | [[35](#_ENREF_35)] |
| 46 | C95M | GS-8373  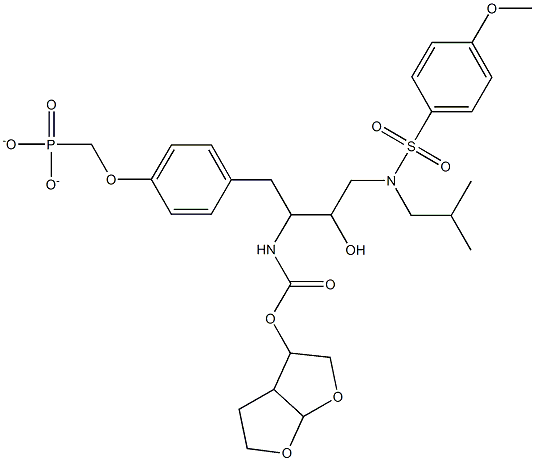 | 2I4D | 0.0158 | [[35](#_ENREF_35)] |
| 46 | C95M | TMC-126  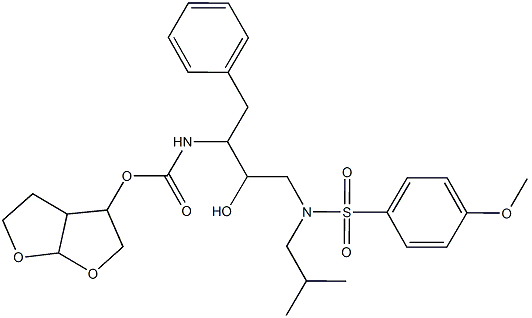 | 2I4U | 0.0097 | [[35](#_ENREF_35)] |
| 47 | no (wild-type sequence) | A-77003  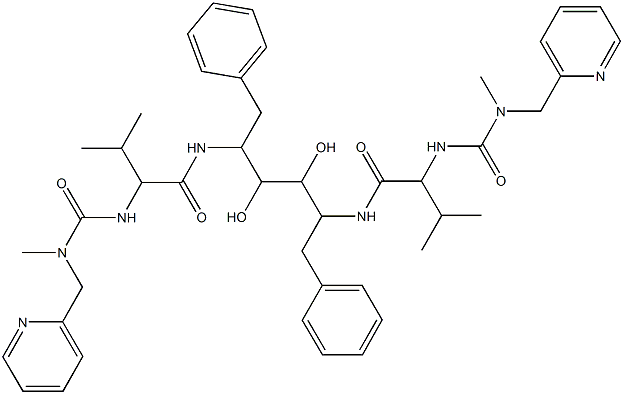 | 1HVI | 0.084 | [[36](#_ENREF_36)] |
| 47 | no (wild-type sequence) | Nelfinavir  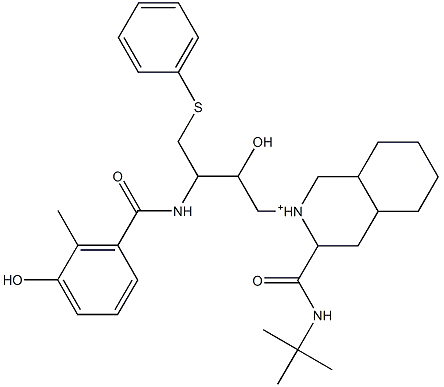 | 1OHR | 2 | [[37](#_ENREF_37)] |
| 47 | no (wild-type sequence) | QN1  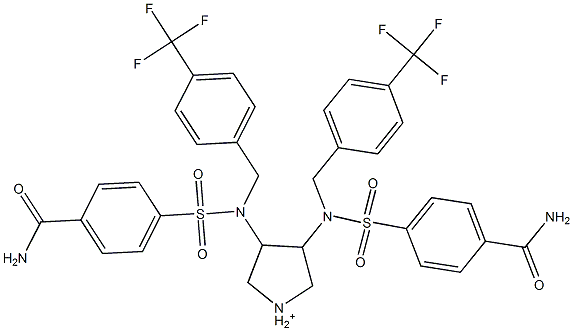 | 2QNN | 70 | [[38](#_ENREF_38)] |
| 47 | no (wild-type sequence) | QN3  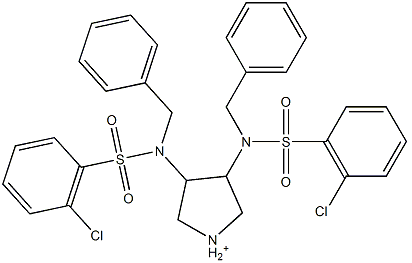 | 2QNQ | 770 | [[38](#_ENREF_38)] |
| 47 | no (wild-type sequence) | AHF  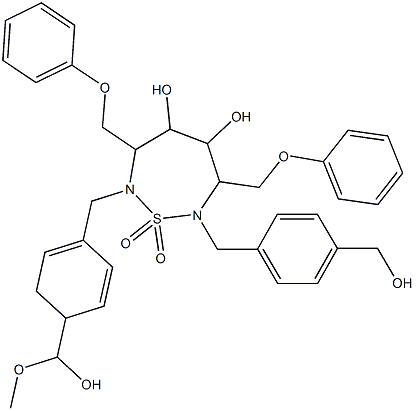 | 1G35 | 7.3 | [[39](#_ENREF_39)] |
| 47 | no (wild-type sequence) | BEE  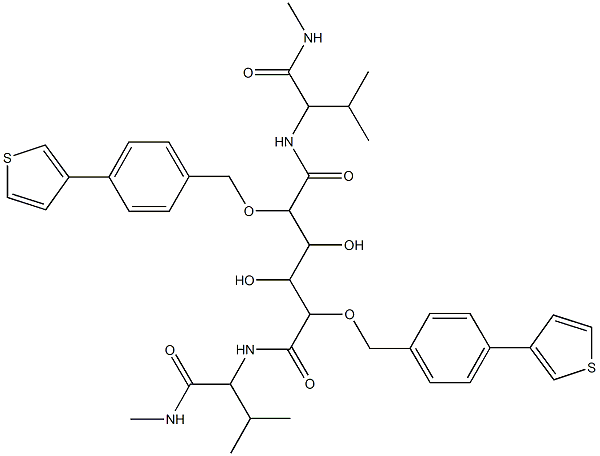 | 1EC1 | 1.2 | [[26](#_ENREF_26)] |
| 47 | no (wild-type sequence) | INU  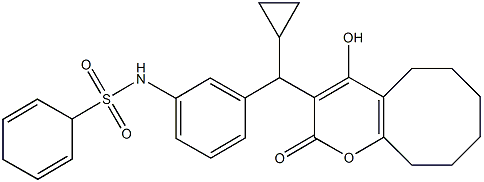 | 7UPJ | 3.2 | [[19](#_ENREF_19)] |
| 47 | no (wild-type sequence) | G4G  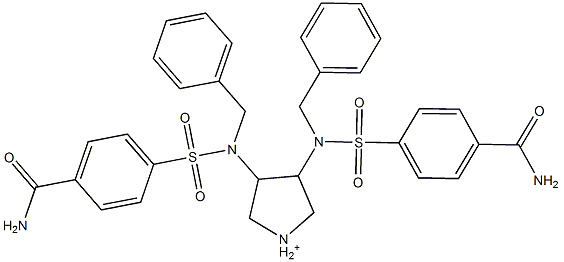 | 2PWR | 260 | [[38](#_ENREF_38)] |
| 47 | no (wild-type sequence) | 190  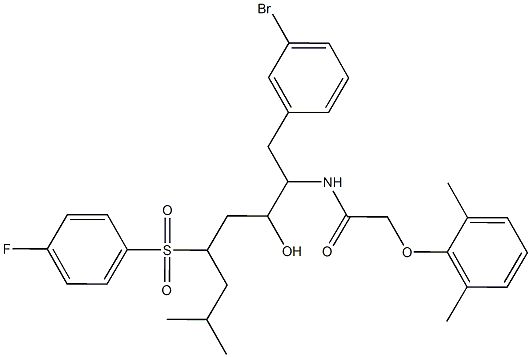 | 1XL5 | 45 | [[40](#_ENREF_40)] |
| 47 | no (wild-type sequence) | Indinavir  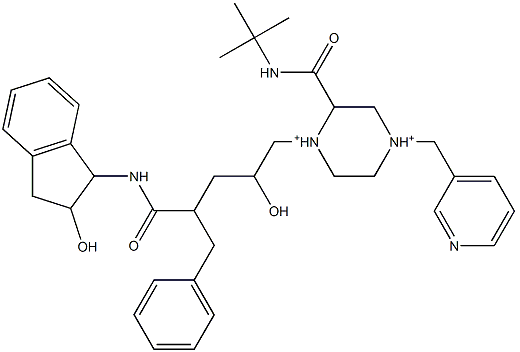 | 1HSG | 0.38 | [[41](#_ENREF_41)] |
| 47 | no (wild-type sequence) | Atazanavir  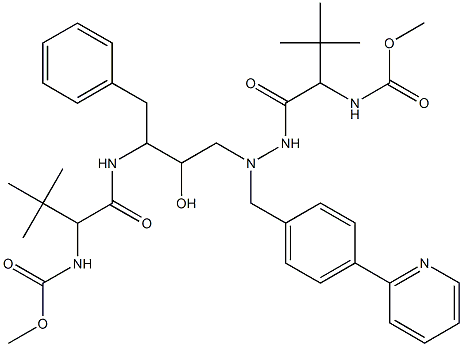 | 2AQU | 0.48 | [[42](#_ENREF_42)] |
| 47 | no (wild-type sequence) | MS3  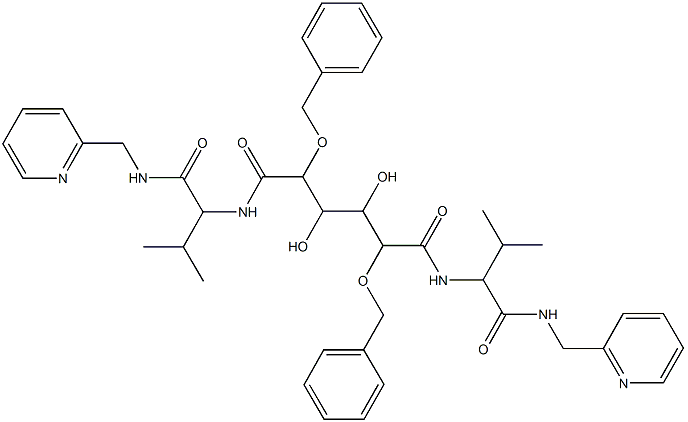 | 1EC3 | 0.92 | [[26](#_ENREF_26)] |
| 47 | no (wild-type sequence) | AH1  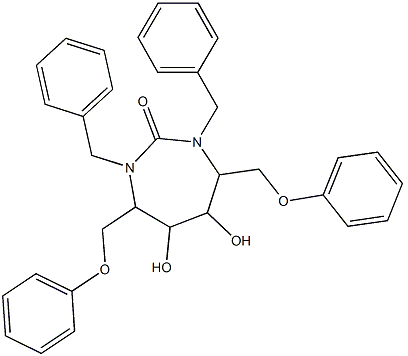 | 1AJX | 12.2 | [[43](#_ENREF_43)] |
| 47 | no (wild-type sequence) | A1A  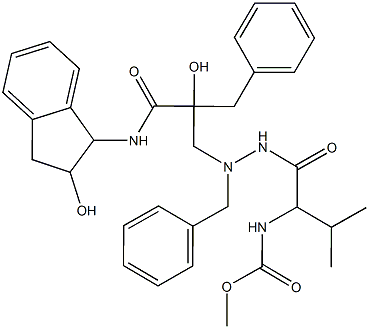 | 2BQV | 9 | [[44](#_ENREF_44)] |
| 47 | no (wild-type sequence) | G3G  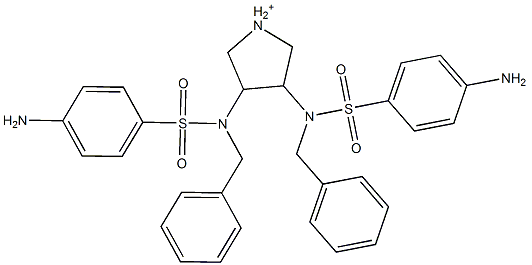 | 2PWC | 270 | [[38](#_ENREF_38)] |
| 47 | no (wild-type sequence) | BED  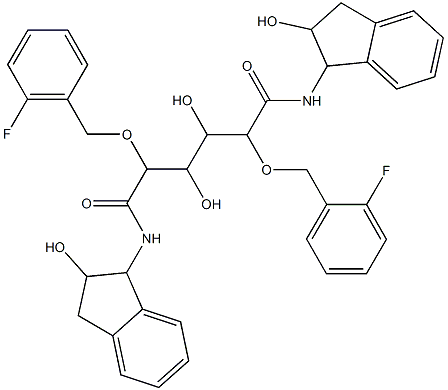 | 1EC0 | 3.2 | [[45](#_ENREF_45)] |
| 47 | no (wild-type sequence) | Amprenavir  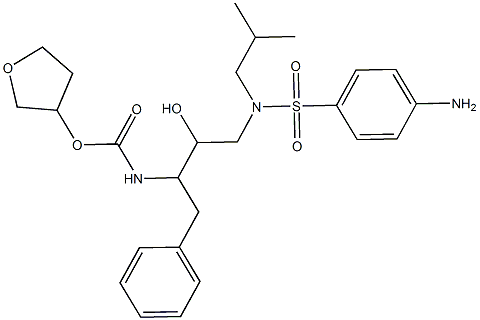 | 1HPV | 0.6 | [[46](#_ENREF_46)] |

## References

1. Ala PJ, Huston EE, Klabe RM, McCabe DD, Duke JL, et al. (1997) Molecular basis of HIV-1 protease drug resistance: structural analysis of mutant proteases complexed with cyclic urea inhibitors. Biochemistry 36: 1573-1580.

2. Ala PJ, Huston EE, Klabe RM, Jadhav PK, Lam PY, et al. (1998) Counteracting HIV-1 protease drug resistance: structural analysis of mutant proteases complexed with XV638 and SD146, cyclic urea amides with broad specificities. Biochemistry 37: 15042-15049.

3. Ala PJ, DeLoskey RJ, Huston EE, Jadhav PK, Lam PY, et al. (1998) Molecular recognition of cyclic urea HIV-1 protease inhibitors. J Biol Chem 273: 12325-12331.

4. Jadhav PK, Ala P, Woerner FJ, Chang CH, Garber SS, et al. (1997) Cyclic urea amides: HIV-1 protease inhibitors with low nanomolar potency against both wild type and protease inhibitor resistant mutants of HIV. J Med Chem 40: 181-191.

5. Hodge CN, Aldrich PE, Bacheler LT, Chang CH, Eyermann CJ, et al. (1996) Improved cyclic urea inhibitors of the HIV-1 protease: synthesis, potency, resistance profile, human pharmacokinetics and X-ray crystal structure of DMP 450. Chem Biol 3: 301-314.

6. Sanches M, Krauchenco S, Martins NH, Gustchina A, Wlodawer A, et al. (2007) Structural characterization of B and non-B subtypes of HIV-protease: insights into the natural susceptibility to drug resistance development. J Mol Biol 369: 1029-1040.

7. Muzammil S, Armstrong AA, Kang LW, Jakalian A, Bonneau PR, et al. (2007) Unique thermodynamic response of tipranavir to human immunodeficiency virus type 1 protease drug resistance mutations. Journal of Virology 81: 5144-5154.

8. Coman RM, Robbins AH, Fernandez MA, Gilliland CT, Sochet AA, et al. (2008) The contribution of naturally occurring polymorphisms in altering the biochemical and structural characteristics of HIV-1 subtype C protease. Biochemistry 47: 731-743.

9. Martin JL, Begun J, Schindeler A, Wickramasinghe WA, Alewood D, et al. (1999) Molecular recognition of macrocyclic peptidomimetic inhibitors by HIV-1 protease. Biochemistry 38: 7978-7988.

10. Nalam MNL, Ali A, Altman MD, Reddy GSKK, Chellappan S, et al. (2010) Evaluating the Substrate-Envelope Hypothesis: Structural Analysis of Novel HIV-1 Protease Inhibitors Designed To Be Robust against Drug Resistance. Journal of Virology 84: 5368-5378.

11. Reddy GSKK, Ali A, Nalam MNL, Anjum SG, Cao H, et al. (2007) Design and synthesis of HIV-1 protease inhibitors incorporating oxazolidinones as P2/P2' ligands in pseudosymmetric dipeptide isosteres. Journal of Medicinal Chemistry 50: 4316-4328.

12. Altman MD, Ali A, Reddy GSKK, Nalam MNL, Anjum SG, et al. (2008) HIV-1 protease inhibitors from inverse design in the substrate envelope exhibit subnanomolar binding to drug-resistant variants. Journal of the American Chemical Society 130: 6099-6113.

13. Reiling KK, Endres NF, Dauber DS, Craik CS, Stroud RM (2002) Anisotropic dynamics of the JE-2147-HIV protease complex: Drug resistance and thermodynamic binding mode examined in a 1.09 angstrom structure. Biochemistry 41: 4582-4594.

14. Liu F, Boross PI, Wang YF, Tozser J, Louis JM, et al. (2005) Kinetic, stability, and structural changes in high-resolution crystal structures of HIV-1 protease with drug-resistant mutations L24I, I50V, and G73S. J Mol Biol 354: 789-800.

15. Wang YF, Tie YF, Boross PI, Tozser J, Ghosh AK, et al. (2007) Potent new antiviral compound shows similar inhibition and structural interactions with drug resistant mutants and wild type HIV-1 protease. Journal of Medicinal Chemistry 50: 4509-4515.

16. Shen CH, Wang YF, Kovalevsky AY, Harrison RW, Weber IT (2010) Amprenavir complexes with HIV-1 protease and its drug-resistant mutants altering hydrophobic clusters. Febs Journal 277: 3699-3714.

17. Liu FL, Kovalevsky AY, Tie YF, Ghosh AK, Harrison RW, et al. (2008) Effect of flap mutations on structure of HIV-1 protease and inhibition by saquinavir and darunavir. Journal of Molecular Biology 381: 102-115.

18. Thaisrivongs S, Skulnick HI, Turner SR, Strohbach JW, Tommasi RA, et al. (1996) Structure-based design of HIV protease inhibitors: Sulfonamide-containing 5,6-dihydro-4-hydroxy-2-pyrones as non-peptidic inhibitors. Journal of Medicinal Chemistry 39: 4349-4353.

19. Skulnick HI, Johnson PD, Aristoff PA, Morris JK, Lovasz KD, et al. (1997) Structure-based design of nonpeptidic HIV protease inhibitors: the sulfonamide-substituted cyclooctylpyramones. J Med Chem 40: 1149-1164.

20. Tie YF, Kovalevsky AY, Boross P, Wang YF, Ghosh AK, et al. (2007) Atomic resolution crystal structures of HIV-1 protease and mutants V82A and I84V with saquinavir. Proteins-Structure Function and Bioinformatics 67: 232-242.

21. Mahalingam B, Wang YF, Boross PI, Tozser J, Louis JM, et al. (2004) Crystal structures of HIV protease V82A and L90M mutants reveal changes in the indinavir-binding site. European Journal of Biochemistry 271: 1516-1524.

22. Tie YF, Boross PI, Wang YF, Gaddis L, Hussain AK, et al. (2004) High resolution crystal structures of HIV-1 protease with a potent non-peptide inhibitor (UIC-94017) active against multi-drug-resistant clinical strains. Journal of Molecular Biology 338: 341-352.

23. Kozisek M, Saskova KG, Rezacova P, Brynda J, van Maarseveen NM, et al. (2008) Ninety-nine is not enough: molecular characterization of inhibitor-resistant human immunodeficiency virus type 1 protease mutants with insertions in the flap region. Journal of Virology 82: 5869-5878.

24. Kozisek M, Bray J, Rezacova P, Saskova K, Brynda J, et al. (2007) Molecular analysis of the HIV-1 resistance development: Enzymatic activities, crystal structures, and thermodynamics of nelfinavir-resistant HIV protease mutants. Journal of Molecular Biology 374: 1005-1016.

25. Swairjo MA, Towler EM, Debouck C, Abdel-Meguid SS (1998) Structural role of the 30's loop in determining the ligand specificity of the human immunodeficiency virus protease. Biochemistry 37: 10928-10936.

26. Andersson HO, Fridborg K, Lowgren S, Alterman M, Muhlman A, et al. (2003) Optimization of P1-P3 groups in symmetric and asymmetric HIV-1 protease inhibitors. European Journal of Biochemistry 270: 1746-1758.

27. Kempf DJ, Marsh KC, Denissen JF, Mcdonald E, Vasavanonda S, et al. (1995) Abt-538 Is a Potent Inhibitor of Human-Immunodeficiency-Virus Protease and Has High Oral Bioavailability in Humans. Proceedings of the National Academy of Sciences of the United States of America 92: 2484-2488.

28. Abdel-Meguid SS, Metcalf BW, Carr TJ, Demarsh P, DesJarlais RL, et al. (1994) An orally bioavailable HIV-1 protease inhibitor containing an imidazole-derived peptide bond replacement: crystallographic and pharmacokinetic analysis. Biochemistry 33: 11671-11677.

29. Weber J, Mesters JR, Lepsik M, Prejdova J, Svec M, et al. (2002) Unusual binding mode of an HIV-1 protease inhibitor explains its potency against multi-drug-resistant virus strains. Journal of Molecular Biology 324: 739-754.

30. Clemente JC, Robbins A, Grana P, Paleo MR, Correa JF, et al. (2008) Design, synthesis, evaluation, and crystallographic-based structural studies of HIV-1 protease inhibitors with reduced response to the V82A mutation. Journal of Medicinal Chemistry 51: 852-860.

31. Saskova KG, Kozisek M, Lepsik M, Brynda J, Rezacova P, et al. (2008) Enzymatic and structural analysis of the I47A mutation contributing to the reduced susceptibility to HIV protease inhibitor lopinavir. Protein Science 17: 1555-1564.

32. Hong L, Zhang XJ, Foundling S, Hartsuck JA, Tang J (1997) Structure of a G48H mutant of HIV-1 protease explains how glycine-48 replacements produce mutants resistant to inhibitor drugs. Febs Letters 420: 11-16.

33. Bottcher J, Blum A, Heine A, Diederich WE, Klebe G (2008) Structural and Kinetic Analysis of Pyrrolidine-Based Inhibitors of the Drug-Resistant Ile84Val Mutant of HIV-1 Protease. Journal of Molecular Biology 383: 347-357.

34. Mahalingam AK, Axelsson L, Ekegren JK, Wannberg J, Kihlstrom J, et al. (2010) HIV-1 Protease Inhibitors with a Transition-State Mimic Comprising a Tertiary Alcohol: Improved Antiviral Activity in Cells. Journal of Medicinal Chemistry 53: 607-615.

35. Cihlar T, He GX, Liu XH, Chen JM, Hatada M, et al. (2006) Suppression of HIV-1 protease inhibitor resistance by phosphonate-mediated solvent anchoring. Journal of Molecular Biology 363: 635-647.

36. Hosur MV, Bhat TN, Kempf DJ, Baldwin ET, Liu B, et al. (1994) Influence of stereochemistry on activity and binding modes for C2 symmetry-based diol inhibitors of HIV-1 protease. Journal of the American Chemical Society 116: 847-855.

37. Kaldor SW, Kalish VJ, Davies JF, Shetty BV, Fritz JE, et al. (1997) Viracept (nelfinavir mesylate, AG1343): A potent, orally bioavailable inhibitor of HIV-1 protease. Journal of Medicinal Chemistry 40: 3979-3985.

38. Blum A, Bottcher J, Heine A, Klebe G, Diederich WE (2008) Structure-guided design of C2-symmetric HIV-1 protease inhibitors based on a pyrrolidine scaffold. J Med Chem 51: 2078-2087.

39. Schaal W, Karlsson A, Ahlsen G, Lindberg J, Andersson HO, et al. (2001) Synthesis and comparative molecular field analysis (CoMFA) of symmetric and nonsymmetric cyclic sulfamide HIV-1 protease inhibitors. Journal of Medicinal Chemistry 44: 155-169.

40. Specker E, Bottcher J, Lilie H, Heine A, Schoop A, et al. (2005) An old target revisited: Two new privileged skeletons and an unexpected binding mode for HIV-protease inhibitors. Angewandte Chemie-International Edition 44: 3140-3144.

41. Chen Z, Li Y, Chen E, Hall D, Darke P, et al. (1994) Crystal structure at 1.9-A resolution of human immunodeficiency virus (HIV) II protease complexed with L-735,524, an orally bioavailable inhibitor of the HIV proteases. J Biol Chem 269: 26344-26348.

42. Clemente JC, Coman RM, Thiaville MM, Janka LK, Jeung JA, et al. (2006) Analysis of HIV-1 CRF_01 A/E protease inhibitor resistance: structural determinants for maintaining sensitivity and developing resistance to atazanavir. Biochemistry 45: 5468-5477.

43. Backbro K, Lowgren S, Osterlund K, Atepo J, Unge T, et al. (1997) Unexpected binding mode of a cyclic sulfamide HIV-1 protease inhibitor. Journal of Medicinal Chemistry 40: 898-902.

44. Ekegren JK, Unge T, Safa MZ, Wallberg H, Samuelsson B, et al. (2005) A new class of HIV-1 protease inhibitors containing a tertiary alcohol in the transition-state mimicking scaffold. Journal of Medicinal Chemistry 48: 8098-8102.

45. Lindberg J, Pyring D, Lowgren S, Rosenquist A, Zuccarello G, et al. (2004) Symmetric fluoro-substituted diol-based HIV protease inhibitors - Ortho-fluorinated and meta-fluorinated P1/P1 '-benzyloxy side groups significantly improve the antiviral activity and preserve binding efficacy. European Journal of Biochemistry 271: 4594-4602.

46. Kim EE, Baker CT, Dwyer MD, Murcko MA, Rao BG, et al. (1995) Crystal-Structure of Hiv-1 Protease in Complex with Vx-478, a Potent and Orally Bioavailable Inhibitor of the Enzyme. Journal of the American Chemical Society 117: 1181-1182.
