# Supplementary material for: Proteochemometric Modeling of the Bioactivity Spectra of HIV-1 Protease Inhibitors by Introducing Protein-Ligand Interaction Fingerprint
Source: PLoS One. 2012 Jul 27;7(7):e41698. doi: 10.1371/journal.pone.0041698 (PMC3407198; doi:10.1371/journal.pone.0041698)
Supplement: Table S2 — Test set used for assessment of the proteochemometric models. (DOCX) [file pone.0041698.s002.docx]

**Table S2.** Test set

| **Protease number** | **Protein** | **Ligand** | **PDBid** | **Ki(nM)** | **Ref** | |
| --- | --- | --- | --- | --- | --- | --- |
| 4 | I3V, C95A | DMP323  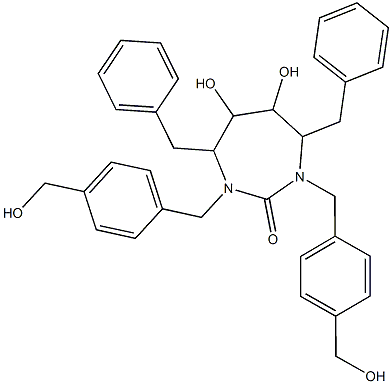 | 1QBS | 0.34 | [[1](#_ENREF_1)] | |
| 4 | I3V, C95A | Q8467  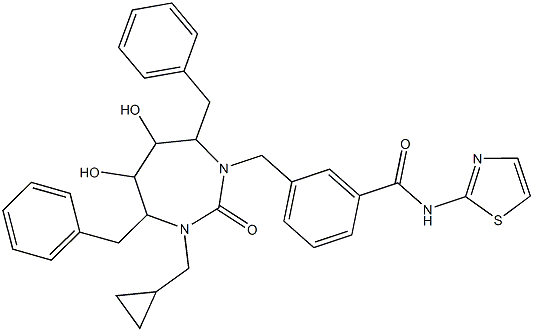 | 1QBU | 0.058 | [[2](#_ENREF_2)] | |
| 5 | Q7K | Atazanavir  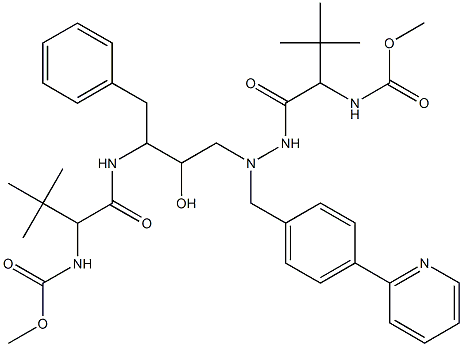 | 2O4K | 0.035 | [[3](#_ENREF_3)] | |
| 5 | Q7K | Tipranavir  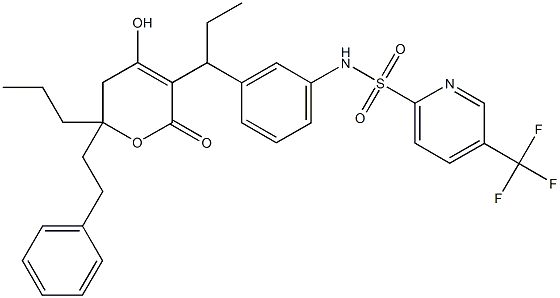 | 2O4P | 0.019 | [[3](#_ENREF_3)] | |
| 9 | Q7K，K14R, L33I，S37N, R41K, L63P, I64V, C67Aba，C95Aba | PI5  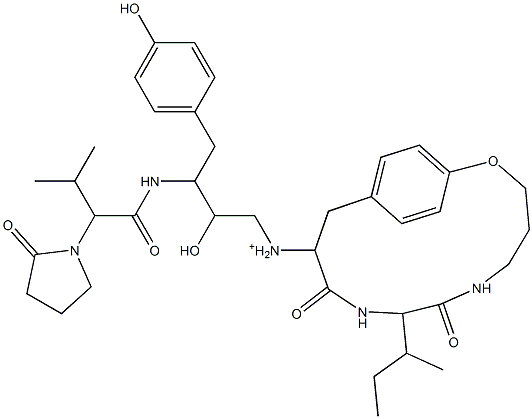 | 1B6K | 1.8 | [[4](#_ENREF_4)] | |
| 9 | Q7K，K14R, L33I，S37N, R41K, L63P, I64V, C67Aba，C95Aba | PI6  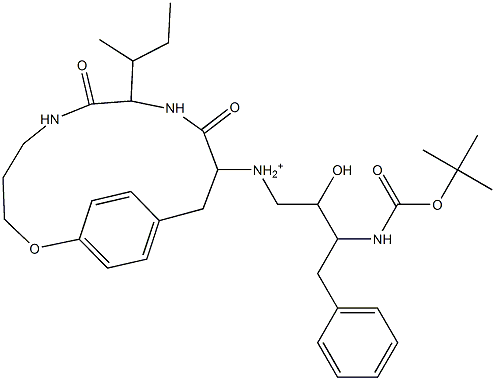 | 1B6M | 4 | [[4](#_ENREF_4)] | |
| 9 | Q7K，K14R, L33I，S37N, R41K, L63P, I64V, C67Aba，C95Aba | PI7  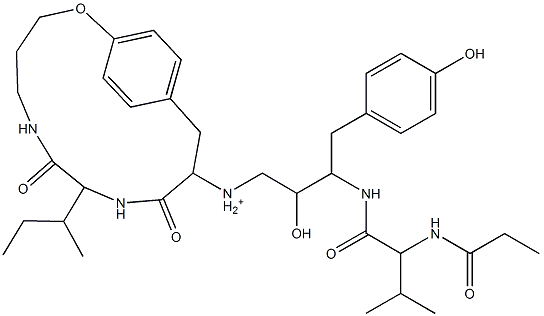 | 1B6P | 3 | [[4](#_ENREF_4)] | |
| 9 | Q7K，K14R, L33I，S37N, R41K, L63P, I64V, C67Aba，C95Aba | HBH  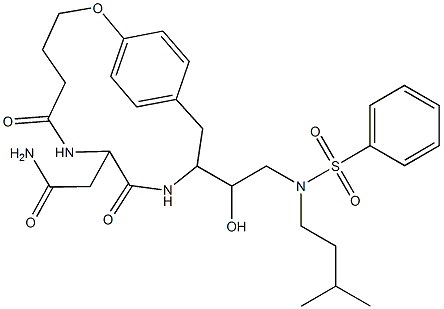 | 1Z1R | 0.6 | [[4](#_ENREF_4)] | |
| 10 | Q7K, K14R, S37N, R41K, L63P | MUI  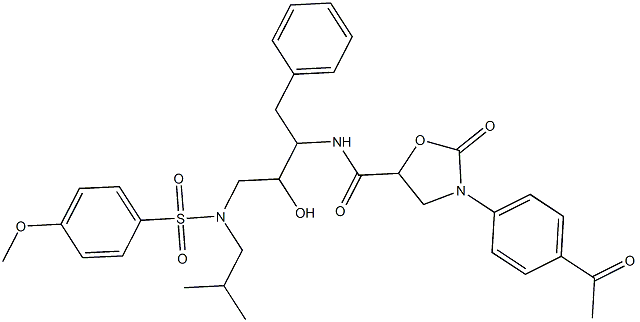 | 2I0A | 0.004 | [[5](#_ENREF_5)] | |
| 10 | Q7K, K14R, S37N, R41K, L63P | MUO  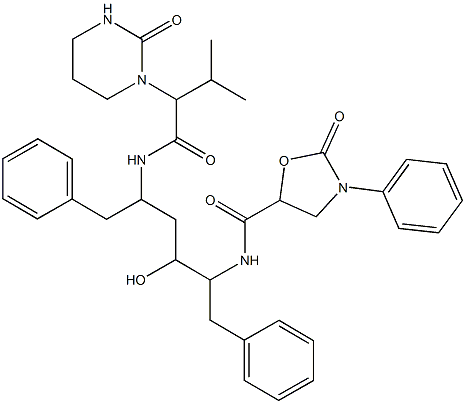 | 2Q55 | 2.04 | [[6](#_ENREF_6)] | |
| 10 | Q7K, K14R, S37N, R41K, L63P | MZ1  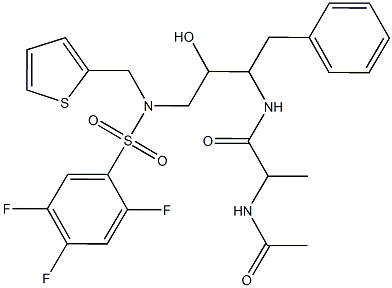 | 2QHY | 33 | [[7](#_ENREF_7)] | |
| 10 | Q7K, K14R, S37N, R41K, L63P | MZ3  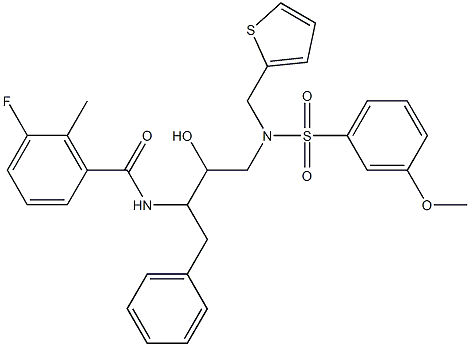 | 2QI0 | 42 | [[7](#_ENREF_7)] | |
| 10 | Q7K, K14R, S37N, R41K, L63P | MZ6  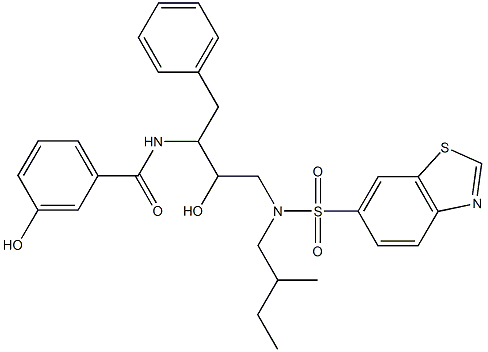 | 2QI4 | 0.036 | [[7](#_ENREF_7)] | |
| 10 | Q7K, K14R, S37N, R41K, L63P | MZ8  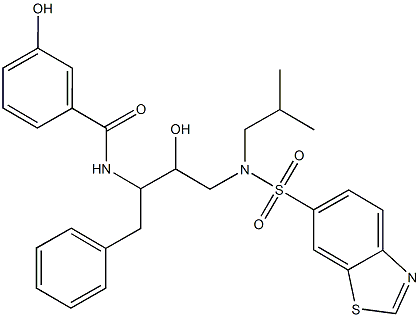 | 2QI6 | 0.027 | | [[7](#_ENREF_7)] |
| 10 | Q7K, K14R, S37N, R41K, L63P | MZ9  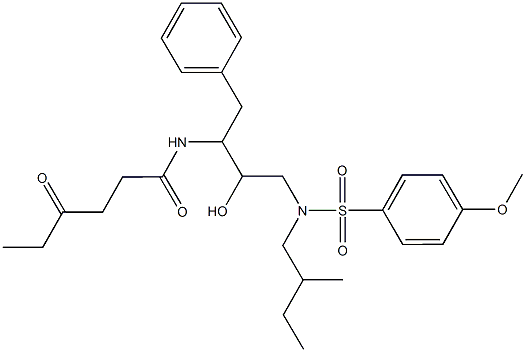 | 2QI7 | 0.062 | | [[7](#_ENREF_7)] |
| 10 | Q7K, K14R, S37N, R41K, L63P | K62  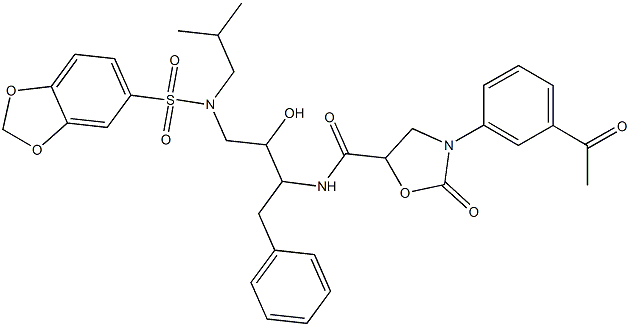 | 3GI5 | 0.006 | | [[8](#_ENREF_8)] |
| 10 | Q7K, K14R, S37N, R41K, L63P | D78  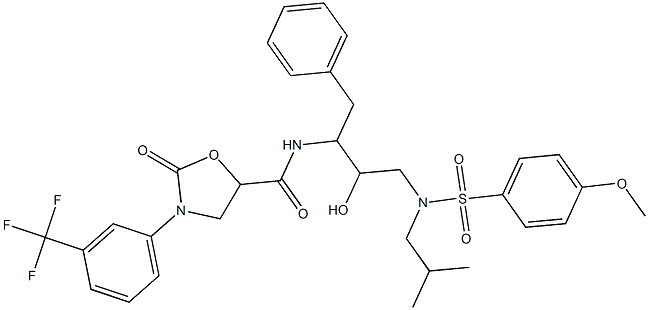 | 3GI6 | 0.006 | | [[8](#_ENREF_8)] |
| 14 | Q7K, D30N, L33I, L63I, C67A, C95A | Darunavir  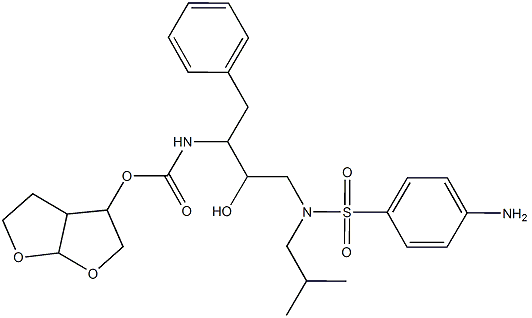 | 2F80 | 6.6 | | [[9](#_ENREF_9)] |
| 17 | Q7K, L33I, I50V, L63I, C67A, C95A | Darunavir  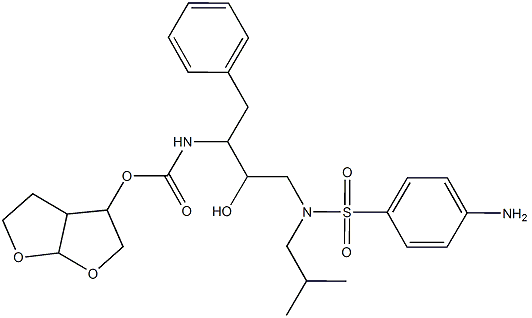 | 2F8G | 2 | | [[9](#_ENREF_9)] |
| 17 | Q7K, L33I, I50V, L63I, C67A, C95A | Amprenavir  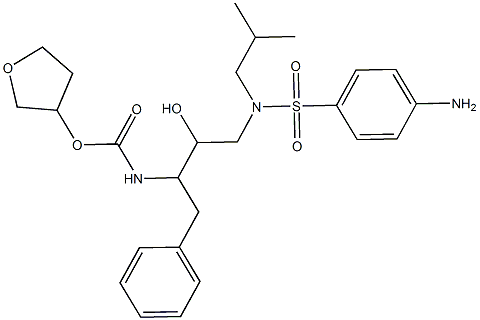 | 3NU5 | 4.5 | | [[10](#_ENREF_10)] |
| 18 | Q7K, L33I, I54M, L63I, C67A, C95A | Darunavir  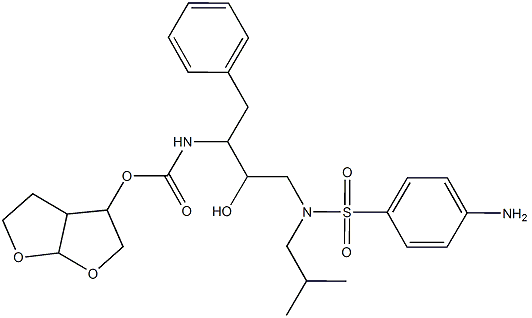 | 3D1Z | 1.6 | | [[11](#_ENREF_11)] |
| 19 | Q7K, L33I, I54V, L63I, C67A, C95A | Darunavir  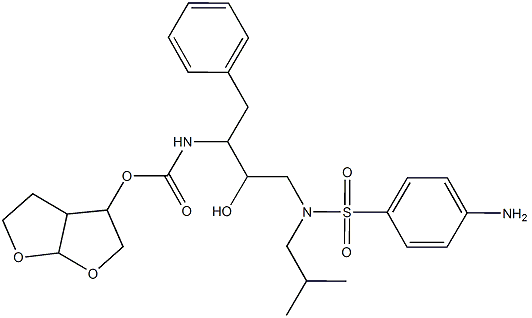 | 3D20 | 5 | | [[11](#_ENREF_11)] |
| 22 | Q7K, L33I, L63I, C67A, V82A, C95A | Darunavir  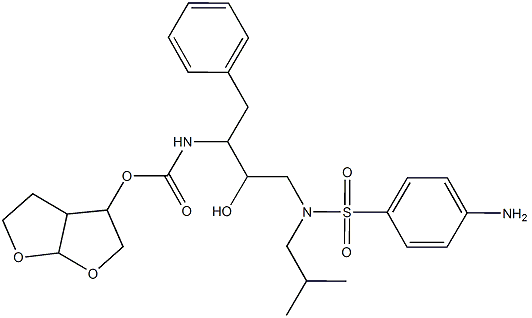 | 2IDW | 1.3 | | [[12](#_ENREF_12)] |
| 22 | Q7K, L33I, L63I, C67A, V82A, C95A | Saquinavir  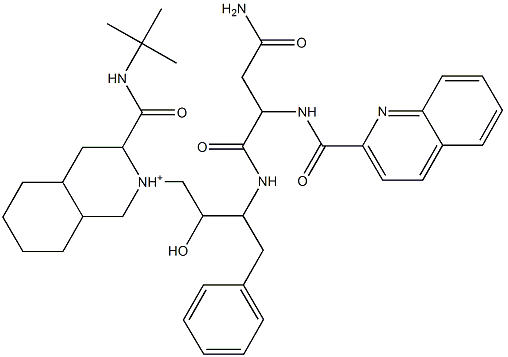 | 2NMY | 4.3 | | [[13](#_ENREF_13)] |
| 23 | Q7K, L33I, L63I, C67A, I84V, C95A | Darunavir  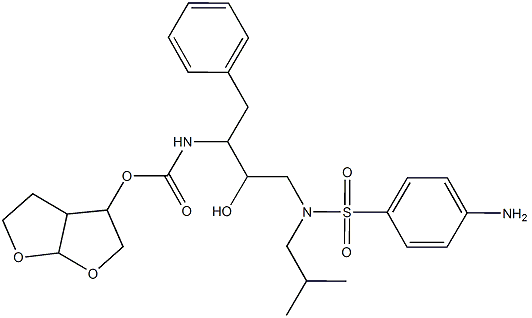 | 2IEO | 3.2 | | [[12](#_ENREF_12)] |
| 23 | Q7K, L33I, L63I, C67A, I84V, C95A | Saquinavir  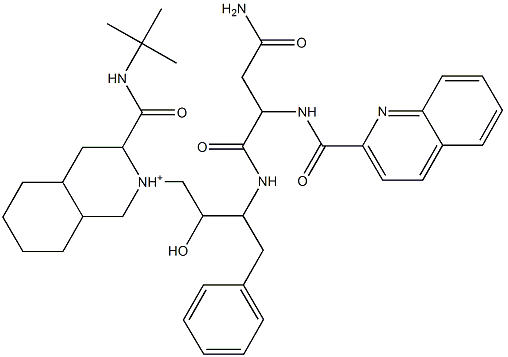 | 2NNP | 4.3 | | [[13](#_ENREF_13)] |
| 23 | Q7K, L33I, L63I, C67A, I84V, C95A | Amprenavir  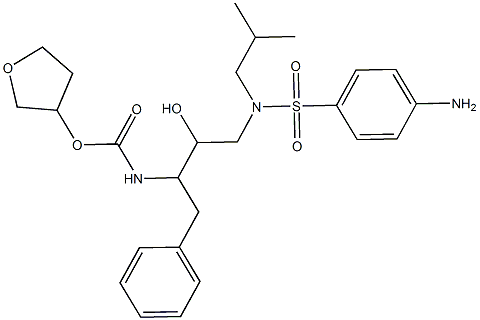 | 3NU9 | 0.9 | | [[10](#_ENREF_10)] |
| 24 | Q7K, L33I, L63I, C67A, L90M, C95A | Darunavir  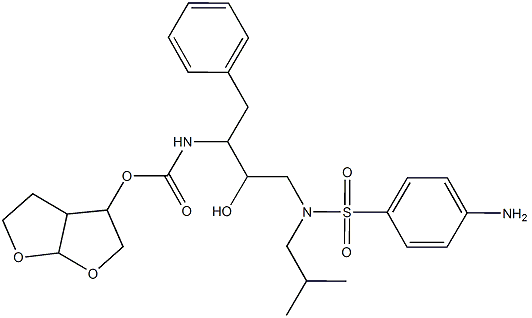 | 2F81 | 0.03 | | [[9](#_ENREF_9)] |
| 25 | Q7K, L33I, L63I, C67A, C95A | 065  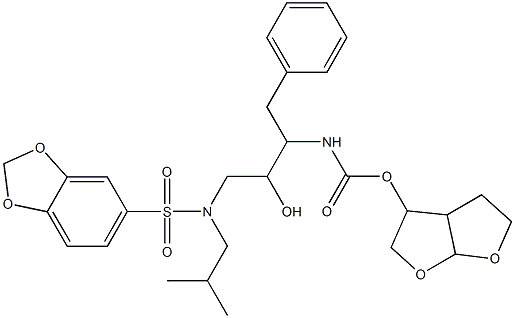 | 2Z4O | 0.27 | | [[14](#_ENREF_14)] |
| 25 | Q7K, L33I, L63I, C67A, C95A | Amprenavir  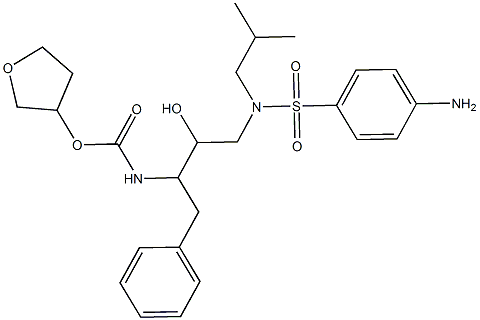 | 3NU3 | 0.15 | | [[10](#_ENREF_10)] |
| 46 | C95M | GS-8374  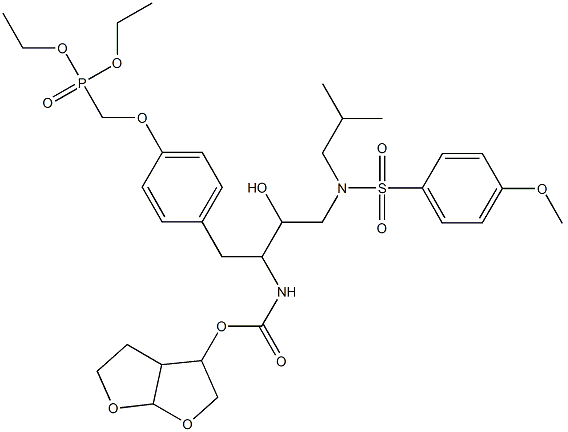 | 2I4W | 0.0152 | | [[15](#_ENREF_15)] |
| 47 | no (wild-type sequence) | NMB  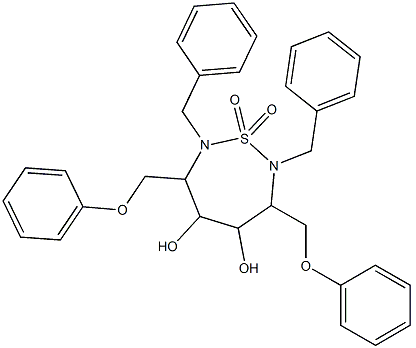 | 1AJV | 19.1 | | [[16](#_ENREF_16)] |
| 47 | no (wild-type sequence) | BEG  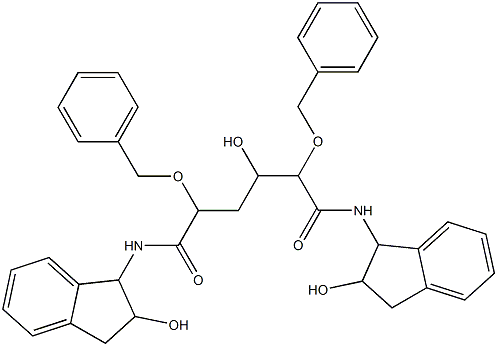 | 1D4I | 1.4 | | [[17](#_ENREF_17)] |
| 47 | no (wild-type sequence) | MSC  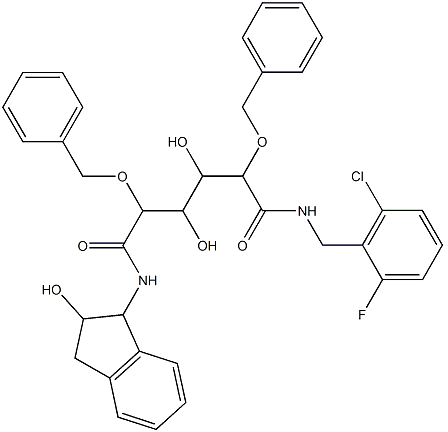 | 1D4J | 4.4 | | [[17](#_ENREF_17)] |
| 47 | no (wild-type sequence) | BEI  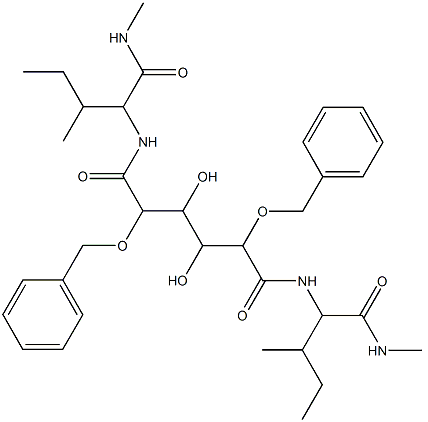 | 1EBW | 0.9 | | [[17](#_ENREF_17)] |
| 47 | no (wild-type sequence) | BEC  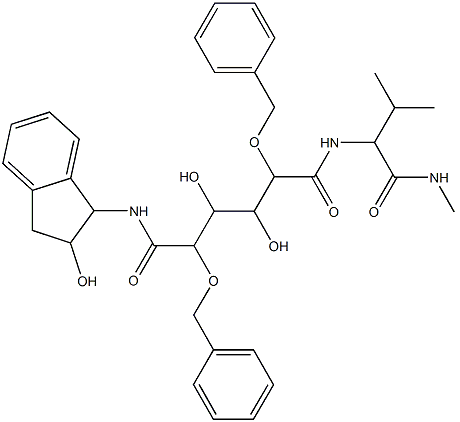 | 1EBZ | 0.4 | | [[17](#_ENREF_17)] |
| 47 | no (wild-type sequence) | BEJ  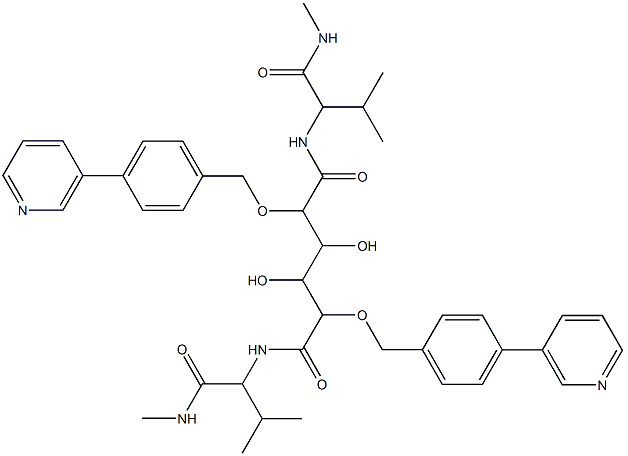 | 1EC2 | 0.1 | | [[17](#_ENREF_17)] |
| 47 | no (wild-type sequence) | NM1  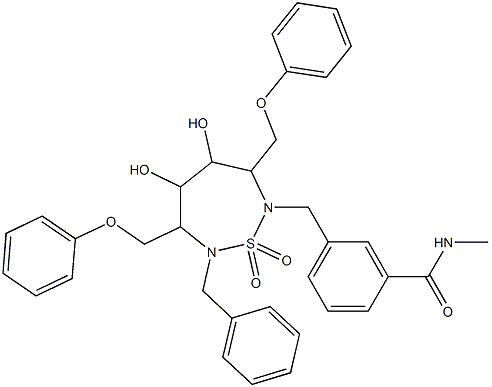 | 1G2K | 11 | | [[18](#_ENREF_18)] |
| 47 | no (wild-type sequence) | A-78791  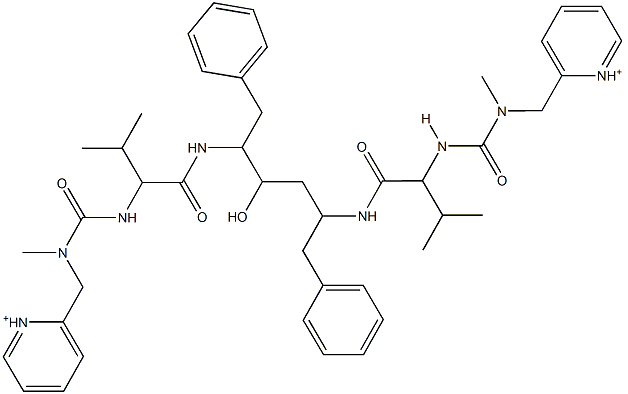 | 1HVJ | 0.035 | | [[19](#_ENREF_19)] |
| 47 | no (wild-type sequence) | A-76928  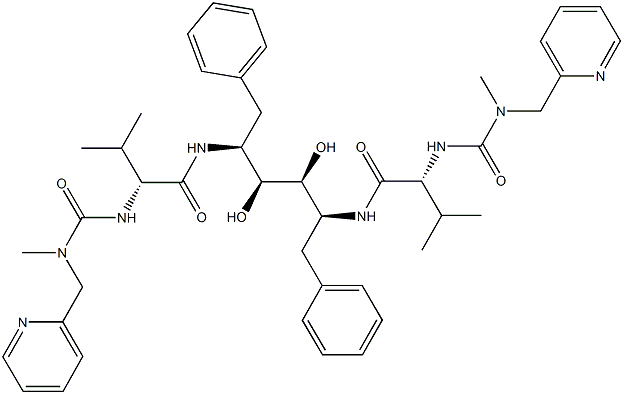 | 1HVK | 0.077 | | [[19](#_ENREF_19)] |
| 47 | no (wild-type sequence) | 1AH  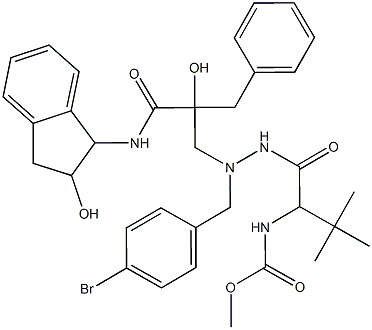 | 2CEJ | 2.4 | | [[20](#_ENREF_20)] |
| 47 | no (wild-type sequence) | 2AH  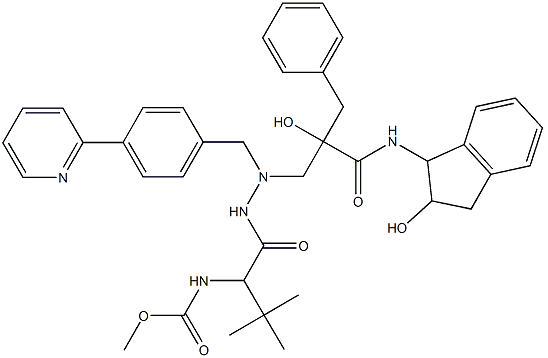 | 2CEM | 12 | | [[20](#_ENREF_20)] |
| 47 | no (wild-type sequence) | 4AH  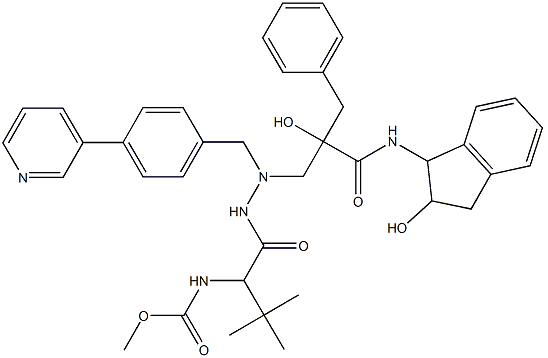 | 2CEN | 5 | | [[20](#_ENREF_20)] |
| 47 | no (wild-type sequence) | G0G  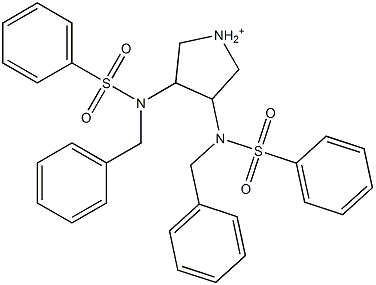 | 2PQZ | 2150 | | [[21](#_ENREF_21)] |
| 47 | no (wild-type sequence) | QN2  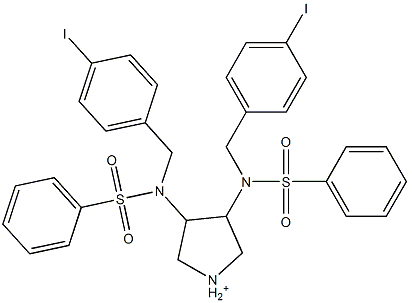 | 2QNP | 390 | | [[21](#_ENREF_21)] |
| 47 | no (wild-type sequence) | HI1  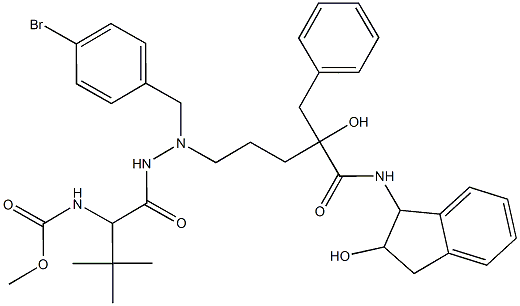 | 2UXZ | 3.3 | | [[22](#_ENREF_22)] |
| 47 | no (wild-type sequence) | 5AH  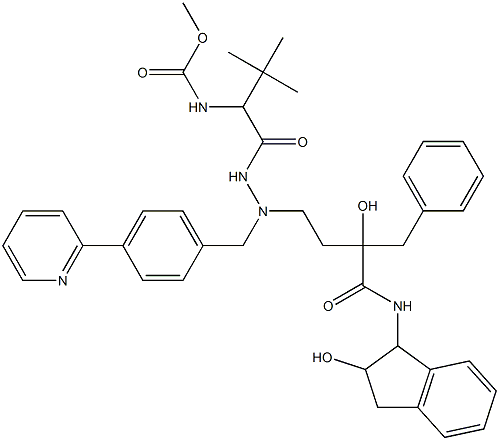 | 2WKZ | 1.7 | | [[23](#_ENREF_23)] |

## References

1. Lam PY, Ru Y, Jadhav PK, Aldrich PE, DeLucca GV, et al. (1996) Cyclic HIV protease inhibitors: synthesis, conformational analysis, P2/P2' structure-activity relationship, and molecular recognition of cyclic ureas. J Med Chem 39: 3514-3525.

2. Jadhav PK, Ala P, Woerner FJ, Chang CH, Garber SS, et al. (1997) Cyclic urea amides: HIV-1 protease inhibitors with low nanomolar potency against both wild type and protease inhibitor resistant mutants of HIV. J Med Chem 40: 181-191.

3. Muzammil S, Armstrong AA, Kang LW, Jakalian A, Bonneau PR, et al. (2007) Unique thermodynamic response of tipranavir to human immunodeficiency virus type 1 protease drug resistance mutations. Journal of Virology 81: 5144-5154.

4. Martin JL, Begun J, Schindeler A, Wickramasinghe WA, Alewood D, et al. (1999) Molecular recognition of macrocyclic peptidomimetic inhibitors by HIV-1 protease. Biochemistry 38: 7978-7988.

5. Ali A, Reddy GSKK, Cao H, Anjum SG, Nalam MNL, et al. (2006) Discovery of HIV-1 protease inhibitors with picomolar affinities incorporating N-aryl-oxazolidinone-5-carboxamides as novel P2 Ligands. Journal of Medicinal Chemistry 49: 7342-7356.

6. Reddy GSKK, Ali A, Nalam MNL, Anjum SG, Cao H, et al. (2007) Design and synthesis of HIV-1 protease inhibitors incorporating oxazolidinones as P2/P2' ligands in pseudosymmetric dipeptide isosteres. Journal of Medicinal Chemistry 50: 4316-4328.

7. Altman MD, Ali A, Reddy GSKK, Nalam MNL, Anjum SG, et al. (2008) HIV-1 protease inhibitors from inverse design in the substrate envelope exhibit subnanomolar binding to drug-resistant variants. Journal of the American Chemical Society 130: 6099-6113.

8. Nalam MNL, Ali A, Altman MD, Reddy GSKK, Chellappan S, et al. (2010) Evaluating the Substrate-Envelope Hypothesis: Structural Analysis of Novel HIV-1 Protease Inhibitors Designed To Be Robust against Drug Resistance. Journal of Virology 84: 5368-5378.

9. Kovalevsky AY, Tie YF, Liu FL, Boross PI, Wang YF, et al. (2006) Effectiveness of nonpeptide clinical inhibitor TMC-114 on HIV-1 protease with highly drug resistant mutations D30N, I50V, and L90M. Journal of Medicinal Chemistry 49: 1379-1387.

10. Shen CH, Wang YF, Kovalevsky AY, Harrison RW, Weber IT (2010) Amprenavir complexes with HIV-1 protease and its drug-resistant mutants altering hydrophobic clusters. Febs Journal 277: 3699-3714.

11. Liu FL, Kovalevsky AY, Tie YF, Ghosh AK, Harrison RW, et al. (2008) Effect of flap mutations on structure of HIV-1 protease and inhibition by saquinavir and darunavir. Journal of Molecular Biology 381: 102-115.

12. Tie YF, Boross PI, Wang YF, Gaddis L, Hussain AK, et al. (2004) High resolution crystal structures of HIV-1 protease with a potent non-peptide inhibitor (UIC-94017) active against multi-drug-resistant clinical strains. Journal of Molecular Biology 338: 341-352.

13. Tie YF, Kovalevsky AY, Boross P, Wang YF, Ghosh AK, et al. (2007) Atomic resolution crystal structures of HIV-1 protease and mutants V82A and I84V with saquinavir. Proteins-Structure Function and Bioinformatics 67: 232-242.

14. Wang YF, Tie YF, Boross PI, Tozser J, Ghosh AK, et al. (2007) Potent new antiviral compound shows similar inhibition and structural interactions with drug resistant mutants and wild type HIV-1 protease. Journal of Medicinal Chemistry 50: 4509-4515.

15. Cihlar T, He GX, Liu XH, Chen JM, Hatada M, et al. (2006) Suppression of HIV-1 protease inhibitor resistance by phosphonate-mediated solvent anchoring. Journal of Molecular Biology 363: 635-647.

16. Backbro K, Lowgren S, Osterlund K, Atepo J, Unge T, et al. (1997) Unexpected binding mode of a cyclic sulfamide HIV-1 protease inhibitor. Journal of Medicinal Chemistry 40: 898-902.

17. Andersson HO, Fridborg K, Lowgren S, Alterman M, Muhlman A, et al. (2003) Optimization of P1-P3 groups in symmetric and asymmetric HIV-1 protease inhibitors. European Journal of Biochemistry 270: 1746-1758.

18. Schaal W, Karlsson A, Ahlsen G, Lindberg J, Andersson HO, et al. (2001) Synthesis and comparative molecular field analysis (CoMFA) of symmetric and nonsymmetric cyclic sulfamide HIV-1 protease inhibitors. Journal of Medicinal Chemistry 44: 155-169.

19. Hosur MV, Bhat TN, Kempf DJ, Baldwin ET, Liu B, et al. (1994) Influence of stereochemistry on activity and binding modes for C2 symmetry-based diol inhibitors of HIV-1 protease. Journal of the American Chemical Society 116: 847-855.

20. Ekegren JK, Ginman N, Johansson A, Wallberg H, Larhed M, et al. (2006) Microwave-accelerated synthesis of P1'-extended HIV-1 protease inhibitors encompassing a tertiary alcohol in the transition-state mimicking scaffold. J Med Chem 49: 1828-1832.

21. Blum A, Bottcher J, Heine A, Klebe G, Diederich WE (2008) Structure-guided design of C2-symmetric HIV-1 protease inhibitors based on a pyrrolidine scaffold. J Med Chem 51: 2078-2087.

22. Wu XY, Oehrngren P, Ekegren JK, Unge J, Unge T, et al. (2008) Two-carbon-elongated HIV-1 protease inhibitors with a tertiary-alcohol-containing transition-state mimic. Journal of Medicinal Chemistry 51: 1053-1057.

23. Mahalingam AK, Axelsson L, Ekegren JK, Wannberg J, Kihlstrom J, et al. (2010) HIV-1 Protease Inhibitors with a Transition-State Mimic Comprising a Tertiary Alcohol: Improved Antiviral Activity in Cells. Journal of Medicinal Chemistry 53: 607-615.
