# Supplementary material for: Proteochemometric Modeling of the Bioactivity Spectra of HIV-1 Protease Inhibitors by Introducing Protein-Ligand Interaction Fingerprint
Source: PLoS One. 2012 Jul 27;7(7):e41698. doi: 10.1371/journal.pone.0041698 (PMC3407198; doi:10.1371/journal.pone.0041698)
Supplement: Table S3 — Removed outliers. (DOCX) [file pone.0041698.s003.docx]

**Table S3.** Outliers

| **Protease number** | **Mutation(s)** | **Ligand** | **PDBid** | | **Ki(nM)** | **Ref** |
| --- | --- | --- | --- | --- | --- | --- |
| 47 | no (wild-type sequence) | BEH  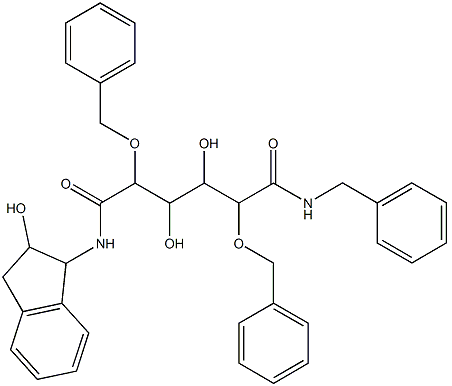 | 1D4H | | 0.1 | [[1](#_ENREF_1)] |
| 4 | I3V, C95A | Q8261  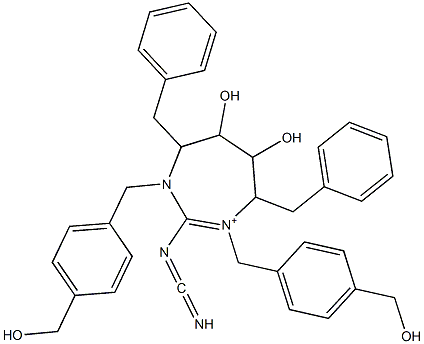 | 1HVH | | 11 | [[2](#_ENREF_2)] |
| 47 | no (wild-type sequence) | A-76889  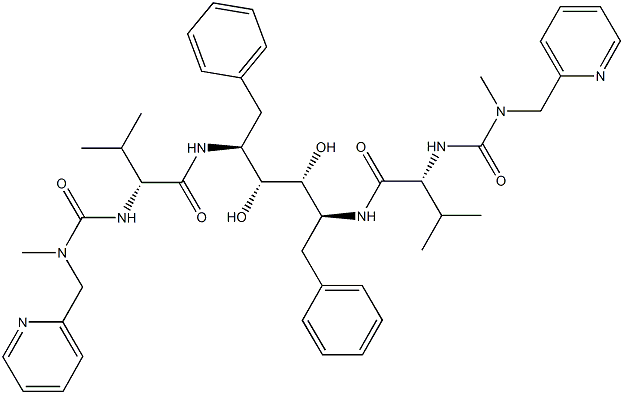 | 1HVL | | 1 | [[3](#_ENREF_3)] |
| 47 | no (wild-type sequence) | Q50  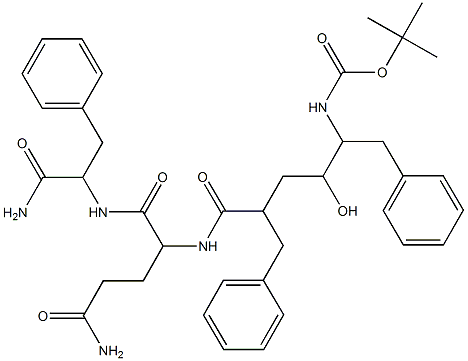 | 1IZH | | 0.02 | [[4](#_ENREF_4)] |
| 2 | I3V, V82F, I84V | DMP323  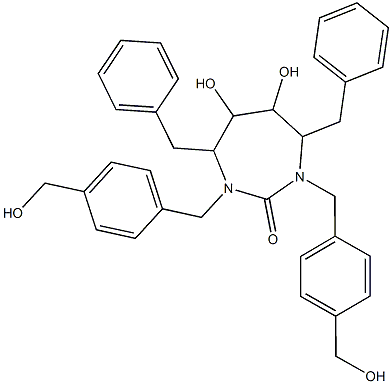 | 1MEU | | 800 | [[5](#_ENREF_5)] |
| 47 | no (wild-type sequence) | Lopinavir  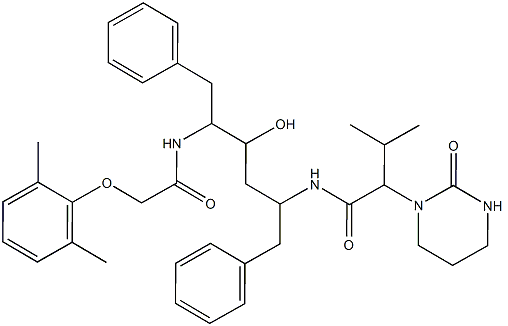 | 1MUI | | 0.0013 | [[6](#_ENREF_6)] |
| 37 | S37N | A-98881  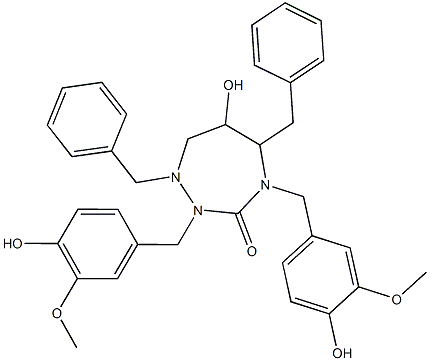 | 1PRO | | 0.005 | [[7](#_ENREF_7)] |
| 30 | K20R, V32I, L33F, M36I, I54V, L63P, A71V, V82A, I84V, L90M | Indinavir  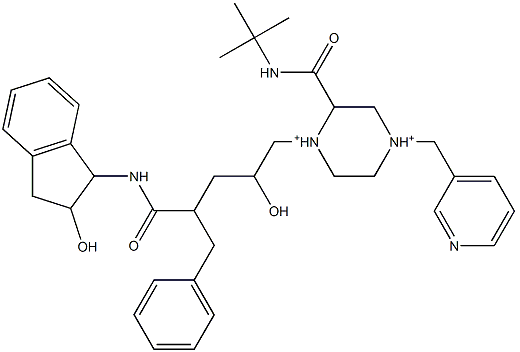 | 1SGU | | 4235 | [[8](#_ENREF_8)] |
| 30 | K20R, V32I, L33F, M36I, I54V, L63P, A71V, V82A, I84V, L90M | Ritonavir  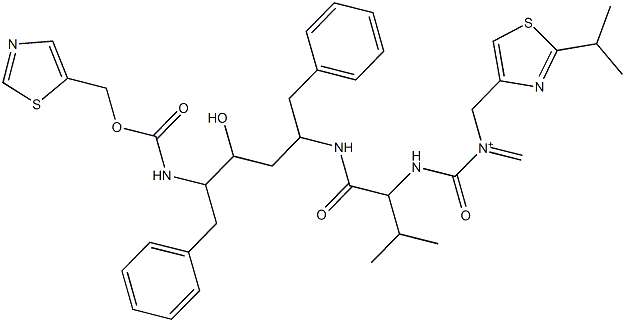 | 1SH9 | | 932 | [[8](#_ENREF_8)] |
| 47 | no (wild-type sequence) | 189  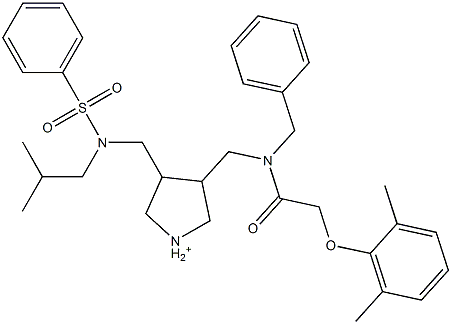 | 1XL2 | | 1500 | [[9](#_ENREF_9)] |
| 47 | no (wild-type sequence) | GW0385  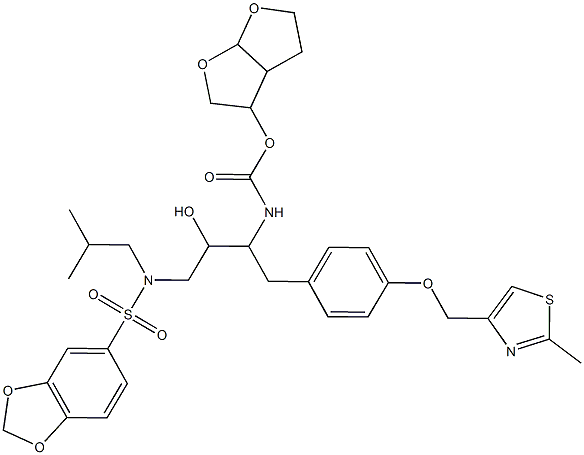 | 2FDE | | 0.000015 | [[10](#_ENREF_10)] |
| 25 | Q7K, L33I, L63I, C67A, C95A | GRL  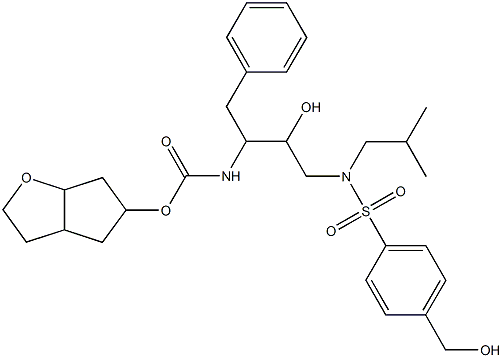 | 2HB3 | | 0.0045 | [[11](#_ENREF_11)] |
| 10 | Q7K, K14R, S37N, R41K, L63P | MUT  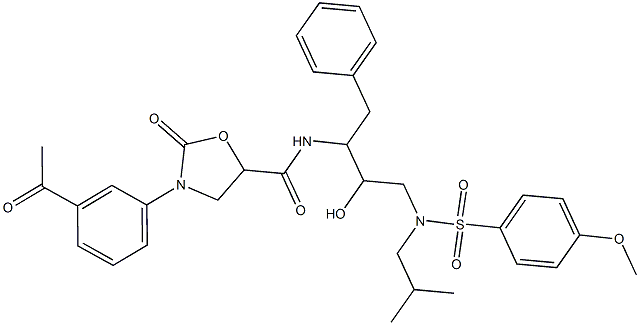 | 2I0D | | 0.0008 | [[12](#_ENREF_12)] |
| 10 | Q7K, K14R, S37N, R41K, L63P | MUU  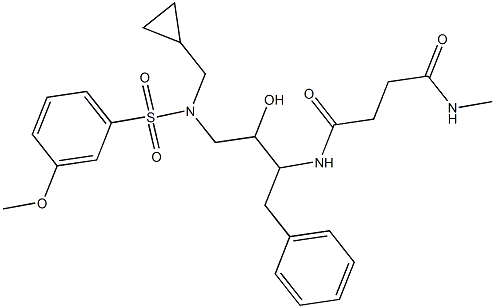 | 2PSU | 24 | | [[13](#_ENREF_13)] |
| 10 | Q7K, K14R, S37N, R41K, L63P | MUV  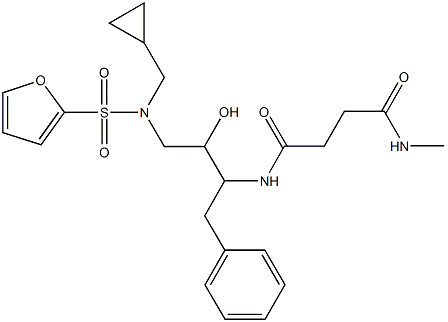 | 2PSV | 58 | | [[13](#_ENREF_13)] |
| 10 | Q7K, K14R, S37N, R41K, L63P | Lopinavir  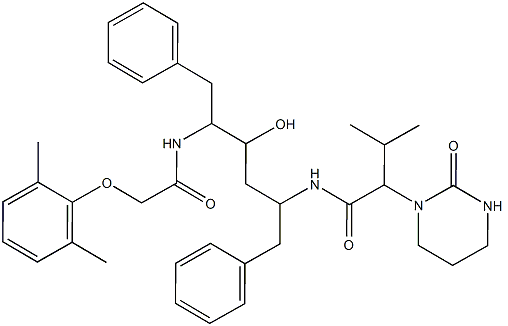 | 2Q5K | 0.005 | | [[14](#_ENREF_14)] |
| 10 | Q7K, K14R, S37N, R41K, L63P | MZ7  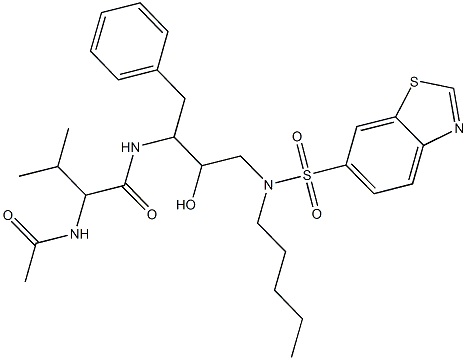 | 2QI5 | 0.014 | | [[15](#_ENREF_15)] |
| 47 | no (wild-type sequence) | HV1  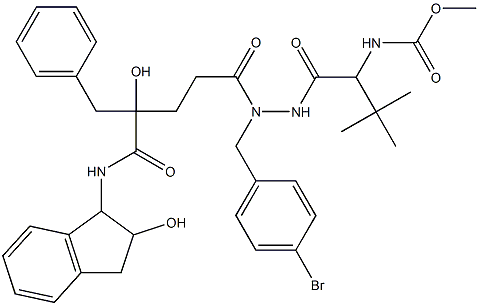 | 2UY0 | 120 | | [[16](#_ENREF_16)] |
| 47 | no (wild-type sequence) | LJG  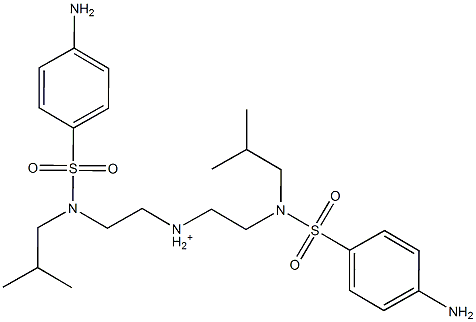 | 3BGB | 900 | | [[17](#_ENREF_17)] |
| 47 | no (wild-type sequence) | LJH  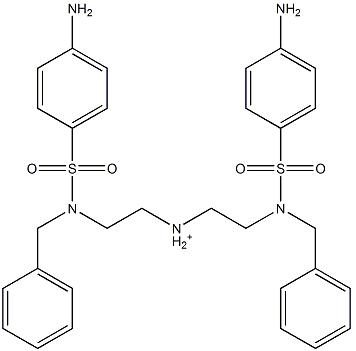 | 3BGC | 9600 | | [[17](#_ENREF_17)] |

## References

1. Andersson HO, Fridborg K, Lowgren S, Alterman M, Muhlman A, et al. (2003) Optimization of P1-P3 groups in symmetric and asymmetric HIV-1 protease inhibitors. European Journal of Biochemistry 270: 1746-1758.

2. Jadhav PK, Woerner FJ, Lam PY, Hodge CN, Eyermann CJ, et al. (1998) Nonpeptide cyclic cyanoguanidines as HIV-1 protease inhibitors: synthesis, structure-activity relationships, and X-ray crystal structure studies. J Med Chem 41: 1446-1455.

3. Hosur MV, Bhat TN, Kempf DJ, Baldwin ET, Liu B, et al. (1994) Influence of stereochemistry on activity and binding modes for C2 symmetry-based diol inhibitors of HIV-1 protease. Journal of the American Chemical Society 116: 847-855.

4. Weber J, Mesters JR, Lepsik M, Prejdova J, Svec M, et al. (2002) Unusual binding mode of an HIV-1 protease inhibitor explains its potency against multi-drug-resistant virus strains. Journal of Molecular Biology 324: 739-754.

5. Ala PJ, Huston EE, Klabe RM, McCabe DD, Duke JL, et al. (1997) Molecular basis of HIV-1 protease drug resistance: structural analysis of mutant proteases complexed with cyclic urea inhibitors. Biochemistry 36: 1573-1580.

6. Stoll V, Qin WY, Stewart KD, Jakob C, Park C, et al. (2002) X-ray crystallographic structure of ABT-378 (lopinavir) bound to HIV-1 protease. Bioorganic & Medicinal Chemistry 10: 2803-2806.

7. Sham HL, Zhao C, Stewart KD, Betebenner DA, Lin S, et al. (1996) A novel, picomolar inhibitor of human immunodeficiency virus type 1 protease. J Med Chem 39: 392-397.

8. Clemente JC, Moose RE, Hemrajani R, Whitford LRS, Govindasamy L, et al. (2004) Comparing the accumulation of active- and nonactive-site mutations in the HIV-1 protease. Biochemistry 43: 12141-12151.

9. Specker E, Bottcher J, Lilie H, Heine A, Schoop A, et al. (2005) An old target revisited: Two new privileged skeletons and an unexpected binding mode for HIV-protease inhibitors. Angewandte Chemie-International Edition 44: 3140-3144.

10. Miller JF, Andrews CW, Brieger M, Furfine ES, Hale MR, et al. (2006) Ultra-potent P1 modified arylsulfonamide HIV protease inhibitors: the discovery of GW0385. Bioorg Med Chem Lett 16: 1788-1794.

11. Ghosh AK, Sridhar PR, Leshchenko S, Hussain AK, Li J, et al. (2006) Structure-based design of novel HIV-1 protease inhibitors to combat drug resistance. J Med Chem 49: 5252-5261.

12. Ali A, Reddy GSKK, Cao H, Anjum SG, Nalam MNL, et al. (2006) Discovery of HIV-1 protease inhibitors with picomolar affinities incorporating N-aryl-oxazolidinone-5-carboxamides as novel P2 Ligands. Journal of Medicinal Chemistry 49: 7342-7356.

13. Chellappan S, Reddy GSKK, Ali A, Nalam MNL, Anjum SG, et al. (2007) Design of mutation-resistant HIV protease inhibitors with the substrate envelope hypothesis. Chemical Biology & Drug Design 69: 298-313.

14. Reddy GSKK, Ali A, Nalam MNL, Anjum SG, Cao H, et al. (2007) Design and synthesis of HIV-1 protease inhibitors incorporating oxazolidinones as P2/P2' ligands in pseudosymmetric dipeptide isosteres. Journal of Medicinal Chemistry 50: 4316-4328.

15. Altman MD, Ali A, Reddy GSKK, Nalam MNL, Anjum SG, et al. (2008) HIV-1 protease inhibitors from inverse design in the substrate envelope exhibit subnanomolar binding to drug-resistant variants. Journal of the American Chemical Society 130: 6099-6113.

16. Wu XY, Oehrngren P, Ekegren JK, Unge J, Unge T, et al. (2008) Two-carbon-elongated HIV-1 protease inhibitors with a tertiary-alcohol-containing transition-state mimic. Journal of Medicinal Chemistry 51: 1053-1057.

17. Blum A, Bottcher J, Sammet B, Luksch T, Heine A, et al. (2008) Achiral oligoamines as versatile tool for the development of aspartic protease inhibitors. Bioorganic & Medicinal Chemistry 16: 8574-8586.
